# Association between methylenetetrahydrofolate reductase C677T polymorphism and cerebral small vessel disease: a systematic review and meta-analysis

**DOI:** 10.3389/fneur.2025.1556535

**Published:** 2025-03-20

**Authors:** Hao-tao Zheng, Wen-wen Lai, Jian-jun Wang, Fan-xin Kong, Hao-bin Cai, Song-jun Lin, Xu Wang, Dong-bin Cai, Min Pi, Xiu-de Qin

**Affiliations:** ^1^Department of Encephalopathy and Phycology, The Fourth Clinical Medical College of Guangzhou University of Chinese Medicine, Shenzhen, China; ^2^Department of Encephalopathy and Phycology, Shenzhen Traditional Chinese Medicine Hospital, Shenzhen, China; ^3^Department of Child Healthcare, Luohu District Maternal and Child Health Hospital, Shenzhen, China; ^4^Department of Preventive Healthcare and Hospital Infection Management, Shenzhen Hospital of Traditional Chinese Medicine, Shenzhen, China

**Keywords:** MTHFR C677T polymorphism, cerebral small vessel disease, meta-analysis, white matter hyperintensities, lacunar infarction, microbleeds

## Abstract

**Objective:**

This systematic review and meta-analysis aimed to evaluate the association between the methylenetetrahydrofolate reductase (5,10-methylenetetrahydrofolate reductase, MTHFR) cytosine (C)677thymine (T) polymorphism and cerebral small vessel disease (CSVD), addressing potential sources of heterogeneity and publication bias.

**Methods:**

An extensive search of databases, including PubMed, the Excerpta Medical Database, and The Cochrane Database of Systematic Reviews, was conducted to identify studies assessing the prevalence of the MTHFR C677T variant associated with CSVD subtypes in humans. Random or fixed effects models were used to accommodate heterogeneity across the study results. Odds ratios (ORs) and weighted mean differences with 95% confidence intervals (CIs) were used for pooled analyses of the relationships between the MTHFR C677T variant associated and CSVD subtypes. Subgroup analyses and assessments of publication bias were performed using Stata software.

**Results:**

Nineteen studies involving 12,441 participants were included. Significant associations were observed across all genetic models: recessive (OR = 1.33; 95%CI = 1.16, 1.52), dominant (OR = 1.25; 95%CI = 1.14, 1.37), allelic (OR = 1.24; 95%CI = 1.14, 1.35), TT vs. CC (OR = 1.42; 95%CI = 1.25, 1.61), and CT vs. CC (OR = 1.20; 95%CI = 1.09, 1.32). Subgroup analyses revealed stronger associations in CSVD-NOS. However, the trim-and-fill method indicated significant publication bias, with adjusted ORs becoming non-significant (recessive model: OR =1.10, 95% CI=0.81, 1.49). Heterogeneity was low to moderate across models (*I*^2^ = 14.2–32.4%).

**Conclusion:**

This study highlights the significant association between MTHFR C677T genotyping and CSVD. Early assessment of MTHFR C677T genotyping during the clinical evaluation of elderly patients may improve patient management and reduce the adverse prognostic impact of the CSVD burden. However, further validation of these findings in large-scale, high-quality prospective studies is required.

**Systematic review registration:**

https://www.crd.york.ac.uk/prospero/; identifier: CRD42023339320.

## 1 Introduction

The incidence of cerebral small vessel disease (CSVD) increases with increasing age, and CSVD is characterized by a range of pathological changes that affect the small arteries, arterioles, venules, and capillaries of the brain. It significantly contributes to age-associated cerebrovascular incidents, including stroke, and is implicated in the decline of vascular-related cognitive function, mood disorders, and mobility challenges (1). Epidemiological data indicate that CSVD occurs in ~50% of all patients with dementia and accounts for approximately 20% of stroke cases worldwide, including a quarter of ischemic stroke cases, approximately one-fifth of patients with stroke have persistent disabilities (2, 3). Traditional risk factors such as smoking, hypertension, diabetes, hyperlipidemia, and obstructive sleep apnea, though extensively investigated, account for only a few CSVD cases. Growing evidence from longitudinal cohort studies highlights the influence of genetic factors on CSVD incidence, suggesting a synergistic interplay between genetic and environmental elements in the etiology of the disease (4, 5). Homocysteine (Hcy), an endogenous amino acid, is associated with endothelial dysfunction and extracellular matrix proliferation, which may lead to vascular damage. Observational research has consistently associated elevated plasma total Hcy (tHcy) levels with an increased CSVD risk (6–8). Furthermore, studies have reinforced the hypothesis that heightened tHcy levels exacerbate vascular pathology (8). Hcy metabolism, which involves folic acid effects, crucial enzymatic activities, and gene polymorphisms, is a key area of study. In particular, the MTHFR gene variant, characterized by a cytosine (C) to thymine (T) substitution at position 677 (rs1801133), was identified as the most significant genetic factor correlated with elevated tHcy levels.

Recent studies have established a correlation between hyperhomocysteinemia (HHcy) and CSVD (7). However, concerns have been raised regarding the reliability of these findings, which is attributed to confounding factors that influence peripheral Hcy levels. Gene detection methodologies offer a strategy to circumvent these confounding effects. Emerging research has revealed an association between a mutation in the Hcy-related MTHFR C677T gene and CSVD pathogenesis (5, 9).

Emerging research has highlighted the association between the MTHFR C677T gene mutation, related to Hcy, and the pathogenesis of CSVD (6). This correlation has attracted research attention, with studies exploring the relationship between the MTHFR C677T polymorphism and both cerebral infarction and vascular cognitive impairment. These investigations have provided preliminary clues regarding the potential link between the MTHFR C677T genotype and CSVD; however, a comprehensive quantitative assessment is currently lacking.

Furthermore, existing research examining the relationship between the MTHFR C677T genotype and specific CSVD subtypes, as well as imaging burden, is limited, and the conclusions are inconsistent (10–13). This inconsistency may be due to differences in sample size, study design, and the rigor of execution, which impact the consistency and reliability of the findings.

Therefore, this meta-analysis aims to clarify the association between the MTHFR C677T gene polymorphism and CSVD by analyzing existing studies. It will assess the impact of the MTHFR C677T gene polymorphism on the risk of CSVD, offering insights for further research into the pathophysiology of CSVD.

## 2 Methods

This study adhered rigorously to the established protocols delineated in the Meta-analysis of Observational Studies in Epidemiology guidelines (MOOSE) (14). Data extraction was conducted in strict accordance with the 2020 Preferred Reporting Items for Systematic Reviews and Meta-Analyses guidelines (15).

### 2.1 Data sources

An exhaustive online search was systematically performed to identify suitable studies using key electronic databases, including PubMed (MEDLINE), the Excerpta Medical Database, and The Cochrane Database of Systematic Reviews. The search spanned the period from the inception of each database until December, 2024. Comprehensive descriptions of the search methodologies and strategies applied to each database are provided in the Supplementary Information. We augmented our study collection by manually reviewing the bibliographies of pertinent research articles. In instances of duplicate publications, the study with the largest cohort was selected for inclusion in the analysis.

### 2.2 Inclusion criteria

The studies were required to meet the criteria as follow: (1) Population (P): Adults were diagnosed with CSVD or its subtypes by neuroimaging (CT or MRI). (2) Intervention/Exposure (I): Genotyping of MTHFR C677T polymorphism with validated methods (such as TaqMan, PCR-RFLP, etc.). (3) Comparison (C): Control groups without CSVD confirmed by neuroimaging (CT or MRI). (4) Outcomes (O): Report the frequencies of cases associated with the MTHFR C677T polymorphism and CSVD. (5) Study Design (S): Observational studies (case-control, cross-sectional, cohort) published in peer-reviewed journals. In cases in which the studies included overlapping participant cohorts, we included only the most extensive dataset available for analysis.

### 2.3 Exclusion criteria

In the present meta-analysis, exclusion criteria were (1) Repeatedly published data; (2) Literature with incomplete data or lacking target indicators; (3) Case reports, prevalence studies, review articles, letters, conference abstracts, and editorials.

### 2.4 Data abstraction

Two independent reviewers meticulously screened the articles and evaluated the titles, abstracts, and full texts of each article. They also assessed the eligibility of the studies and systematically extracted relevant information and data. In instances where a consensus was not reached, an independent expert was consulted for resolution. The inclusion criteria for the trials were studies that provided data on specific outcomes, including CSVD subtypes and the prevalence of the MTHFR C677T genotype in both the case and control groups. Three independently investigators extracted data from the selected articles. The extracted data included the publication year, study design, sample size, demographic details, CSVD subtype, and outcome indicators.

### 2.5 Evaluation of the quality of the studies

The Newcastle-Ottawa Quality Assessment Scale was used to appraise the methodological quality of observational case-control studies. This scale evaluates three primary domains: the selection process of cases and controls, comparability between groups, and accuracy of exposure or outcome ascertainment, with a scoring range of 0–8 points. A higher score indicates superior methodological quality.

The quality of the selected studies was independently assessed by two investigators, and the outcomes of these assessments were meticulously recorded in a dedicated spreadsheet. Discrepancies in ratings were reconciled either through consensus or through the intervention of a third reviewer. An average NOS score of 7.1 indicates the high methodological quality of the included studies. The level of agreement between the raters was remarkably high, as indicated by a Cohen's kappa coefficient of 0.694 (95% confidence interval [CI]: 0.42–0.97).

### 2.6 Outcomes measures

Studies were considered eligible for inclusion if they presented data addressing specific outcomes, such as CSVD subtypes and the prevalence of the MTHFR C677T genotype in both case and control cohorts. The analysis primarily aimed to discern variations in the aggregated prevalence of individuals exhibiting diverse MTHFR C677T polymorphisms, with a comparison between CSVD and control groups and among different neuroimaging CSVD subtypes.

CSVD encompasses a range of diseases identified through clinical and imaging observations, resulting from pathological processes that affect cerebral arterioles, capillaries, and venules. Neuroimaging features of SVD include recent small subcortical infarcts, lacunes, white matter hyperintensities, perivascular spaces, microbleeds, and brain atrophy (16).

Lacunar infarction (LI): LI referred to as lacunar strokes or lacunar syndromes, represent a morphological entity characterized by small areas of cerebral infarction. These infarctions typically range in size from 0.5 to 15.0 mm in diameter and are predominantly located in deep cerebral regions, including the subcortical white matter, basal ganglia, and pontine base. The central liquefaction in lacunar infarctions results in MRI features that mimicking cerebrospinal fluid, characterized by hypointensity on T1-weighted imaging (T1WI), hyperintensity on T2-weighted imaging (T2WI), and hypointensity on fluid-attenuated inversion recovery (FLAIR) sequences, occasionally surrounded by a hyperintense rim. On diffusion-weighted imaging (DWI), these infarctions exhibit isointense or hypointense signals, and hyperintensity on T2^*^/susceptibility-weighted imaging (SWI), with diameters typically ranging from 3 to 15 mm (17).

White Matter Hyperintensities (WMH): Vasogenic WMHs exhibit a range of pathological features, including demyelination, gliosis, axonal loss, and oligodendrocyte depletion, with confluent lesions spanning broader regions. The magnetic resonance imaging characteristics of periventricular and deep WMHs encompass thickening of vessel walls, enlargement of the perivascular space (PVS), reduced vascular density, and increased vascular tortuosity. These hyperintensities are observed on T2WI and FLAIR sequences as foci of variable size, with isointense or hypointense signals on T1WI and DWI, and hyperintensity on SWI, which is distinct from the cerebrospinal fluid signal associated with cavitation. They are frequently bilaterally symmetrical and situated adjacent to the lateral ventricles and within the deep white matter (17).

Cerebral Microbleeds (CMBs): Cerebral microbleeds are commonly identified as clusters of iron-laden pigment granules surrounded by macrophages, with associated perilesional tissue alterations such as rarefaction, destruction, and gliosis. These granules may also be present within the PVS or brain tissue without associated tissue damage. On MRI, microbleeds appear as small, round hypointensities on SWI, with isointense signals on T1WI, T2WI, FLAIR, and DWI sequences, typically measuring 2 to 5 mm in diameter, although they can occasionally extend up to 10 mm (17).

### 2.7 Statistical analysis

The chi-square test was used to determine whether the genotype distribution in the control group adhered to the Hardy–Weinberg equilibrium (HWE). Odds ratios (ORs) with 95% confidence intervals (CIs) were extracted or calculated to evaluate the relationship between the MTHFR C677T polymorphism and CSVD subtypes. Meta-analyses were conducted under five genetic models: recessive model (TT vs. CT + CC), dominant model (TT + CT vs. CC), allelic model (T vs. C), homozygote contrast (TT vs. CC), and heterozygote genotype vs. wild homozygote genotype contrast (CT vs. CC). Log-transformed ORs and CIs were pooled using a random-effects model to account for anticipated heterogeneity across studies. The extent of heterogeneity among study results was quantified using *I*^2^ statistics. An *I*^2^ value of 0%, < 25%, 25%−50%, and >50% indicated the absence of heterogeneity, low heterogeneity, moderate heterogeneity, and high heterogeneity, respectively. A fixed-effects model was applied only if both *I*^2^ ≤ 50% and Cochran's Q-test P >0.10 indicated homogeneity. Subgroup analyses and meta-regression: Predefined subgroups included disease subtype (CSVD-NOS, WMH, LI, CMBs), ethnicity (Asian, European), imaging criteria (MRI, CT/MRI), and HWE compliance (yes/no). Sensitivity analysis was performed to assess the impact of individual studies on the aggregate OR by sequentially excluding one study at a time and recalculating the combined ORs for the remaining studies. Egger's and Begg's tests were conducted to evaluate potential publication bias. These tests are recognized as standard procedures in meta-analyses for examining funnel plot asymmetry. If bias was detected, the trim-and-fill method adjusted for potential missing studies. Two studies (18, 19) were excluded from genotype-specific analyses due to incomplete reporting of CC/CT frequencies. Despite email correspondence with authors, raw data were unavailable. All analyses were performed in Stata 13.0, with statistical significance at two-tailed P < 0.05.

## 3 Results

### 3.1 Study selection

**Figure 1:**
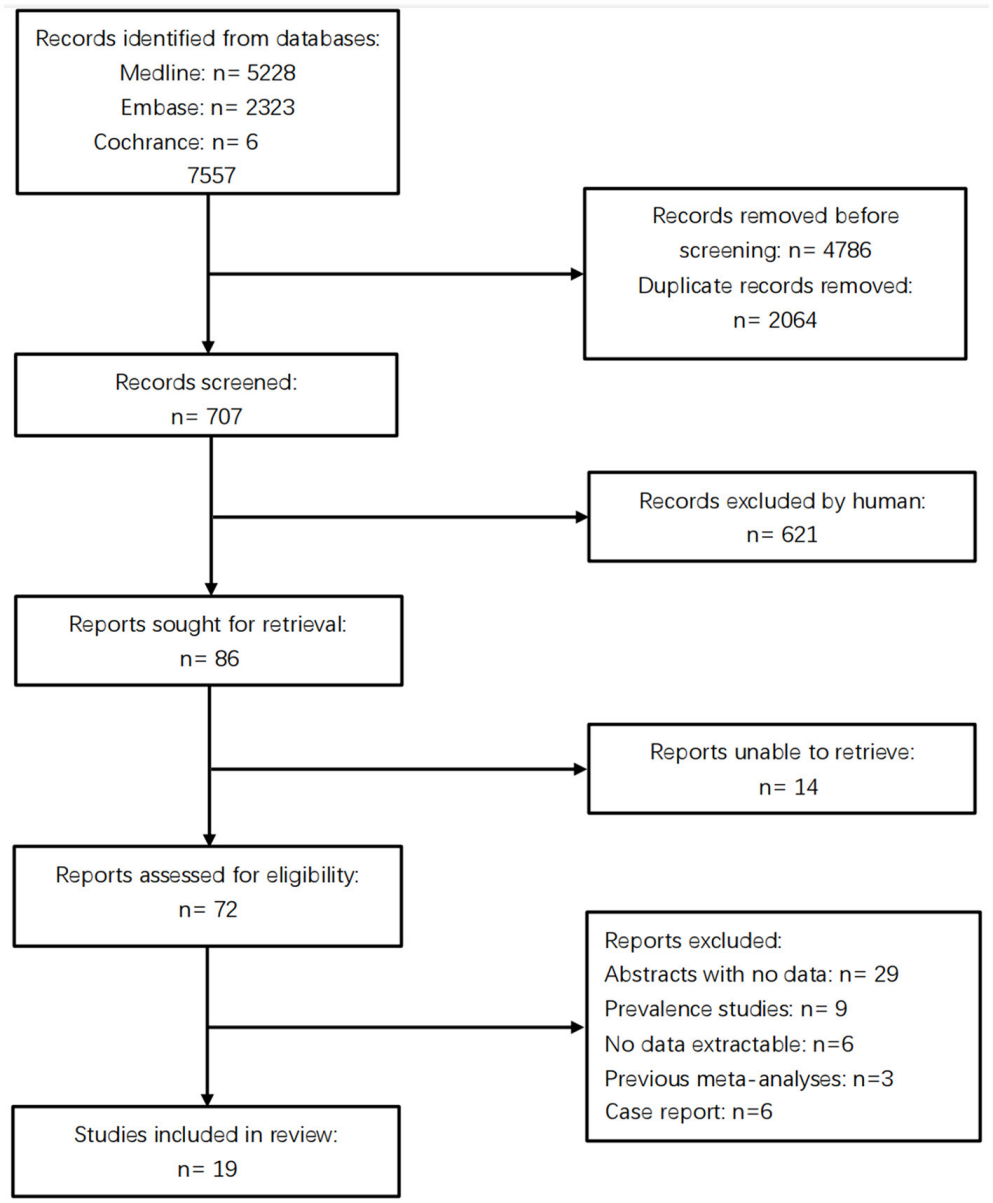
The prism flowchart indicating the articles' screening and exclusion process up to final inclusion in the qualitative and quantitative analyses.

The database search yielded 7,557 citations. Following the screening process detailed in Figure 1, 19 full-length articles met inclusion criteria for detailed consideration. Consequently, the meta-analysis included 12,441 participants (3,676 cases and 8,765 controls) across four cerebrovascular phenotypes: 1,143 with CSVD-NOS, 1,125 with WMH, 1,247 with LI, 161 with CMBs. Table 1 presents a detailed overview of studies focusing on the association between the MTHFR C677T genotype and the clinical manifestations of CSVD.

**Table 1:** Demographics and clinical features of the case-control studies.

**References**	**Region (race)**	**subtype**	**No**.	**Mean age (SD)**	**%Male**	**%Hypertensive**	**CT/MRI**	**Nos**
Hassan (4)	UK(E)	SVD	172	67 (10)	59	76	MRI	5
		CTR	172	66 (10)	58	42	MRI	
Yeh et al. (31)	China Taiwan (AS)	SVD	231	45 (6)	75	58	Both	6
		CTR	200	46 (8)	84	13	No	
Choi et al. (20)	Korea (AS)	SVD	72	61 (11)	54	65	MRI	6
		CTR	198	61 (11)	56	39	No	
Szolnoki et al. (30)	Hungary (E)	SVD	123	63 (8)	53	48	Both	8
		CTR	263	55 (13)	52	18	Both	
Pavlovic et al. (28)	Serbia (E)	SVD	95	60 (11)	58	NR	MRI	8
		CTR	41	58 (11)	46	NR	MRI	
Luo et al. (25)	China (AS)	SVD	221	66 (12)	62	58	Both	6
		CTR	774	52 (17)	54	19	NR	
Yuan et al. (5)	China (AS)	SVD	163	63 (7)	58	33	NR	6
		CTR	326	63 (7)	58	20	NR	
Chen et al. (33)	China (AS)	SVD	191	67 (9)	81	90	MRI	8
		No-SVD	182	67 (8)	84	59	MRI	
Notsu et al. (27)	Japan (AS)	SBI	74	66 (1)	68	80	MRI	7
		CTR	214	60 (1)	58	24	NR	
Kohara et al. (23)	Japan(AS)	WMH	496	68 (8)	52	51	MRI	8
		CTR	1225	55 (10)	51	27	NR	
Szolnoki et al. (18)	Hungary (E)	WMH	315	69 (14)	55	48	MRI	8
		CTR	646	58 (11)	54	27	MRI	
Szolnoki et al. (34)	Hungary (E)	WMH	198	65 (9)	47	70	MRI	7
		CTR	235	54 (13)	46	19	MRI	
Li et al. (24)	China (AS)	WMH	104	67 (8)	59	76	MRI	8
		CTR	74	59 (7)	58	22	MRI	
You et al. (32)	China (AS)	WMH	60	67 (9)	45	32	MRI	8
		CTR	30	65 (8)	43	50	MRI	
Kawamoto et al. (22)	Japan (AS)	Li	38	78 (8)	59	62	CT	7
		CTR	241	76 (7)	49	42	NR	
Shen et al. (29)	China (AS)	Li	513	61 (9)	63	60	Both	7
		CTR	1,832	60 (9)	57	26	NR	
Mao et al. (26)	China (AS)	Li	159	67 (11)	63	85	Both	7
		CTR	198	65 (11)	55	48	Both	
Feng et al. (21)	China (AS)	Li	415	61 (9)	63	61	Both	8
		CTR	1,263	60 (8)	61	27	Both	
Yoo et al. (19)	Korea (AS)	CMB	161	77 (8)	39	70	MRI	7
		CTR	658	73 (10)	29	49	MRI	

No., number; SVD, small vessel disease; CTR, controls; SBI, silent brain infarction; CMB, cerebral microbleeds; Ls, lacunar stroke; Li, lacunar infarction; WMH, white matter hyperintensity; UK, United Kingdom; US, United States; AS, Asia; E, European; NR, not reported; MRI, magnetic resonance imaging; SD, standard deviation; CT, computed tomography; NOS, Newcastle-Ottawa scale quality assessment.

### 3.2 Study characteristics

The analysis incorporated 19 case-control studies (4, 5, 18–33) (Table 1), including 8 in CSVD-NOS (4, 5, 20, 25, 28, 30, 31, 33), 6 in WMH (18, 23, 24, 27, 32, 34), 4 in LI (21, 22, 26, 29), and 1 in CMBs (19). Sample sizes ranged from 90 to 2,345 participants (total *N* = 12,441), with mean ages of 40–78 years. The geographical distribution of the studies was as follows: 14 studies were from Asia (China: 9; Japan: 3; South Korea: 2), and 5 studies were from Europe (Hungary: 3; Serbia: 1; United Kingdom: 1). The imaging modalities comprised MRI-only protocols (10 studies) (18–20, 23, 24, 27, 28, 32, 34), and CT/MRI protocols (nine studies) (4, 5, 21, 22, 25, 26, 29–31, 33). Thirteen studies conformed to HWE, while six showed deviations (Table 2). Eighteen studies reported the prevalence of hypertension, while only four studies reported levels of homocysteine (19, 24, 32).

**Table 2:** Distribution of genotypes in the individual studies.

**References**	**Case group**					**Control group**					**HWE for control**	**MAF**
	**CC**	**CT**	**TT**	**C**	**T**	**CC**	**CT**	**TT**	**C**	**T**		
Hassan (4)	66	71	33	203	137	81	73	16	235	105	0.94	0.31
Yeh et al. (31)	51	50	7	152	64	111	85	4	307	93	0.01	0.23
Choi et al. (20)	20	32	20	72	72	73	100	25	246	150	0.30	0.38
Szolnoki et al. (30)	60	43	20	163	83	148	89	26	385	141	0.03	0.27
Pavlovic et al. (28)	36	42	17	114	76	15	20	6	50	32	0.87	0.39
Luo et al. (25)	118	86	17	322	120	423	299	52	1,145	403	0.93	0.26
Yuan et al. (5)	28	69	66	125	201	106	113	107	325	327	3.05	0.50
Chen et al. (33)	30	86	75	146	236	40	97	45	177	187	0.37	0.51
Notsu et al. (27)	23	33	18	79	69	86	92	31	264	154	0.43	0.37
Kohara et al. (23)	168	239	89	575	417	455	571	199	1,481	969	0.38	0.40
Szolnoki et al. (18)	271(CC+CT)		44(TT)			557(CC+CT)		89(TT)				
Szolnoki et al. (34)	70	98	30	238	158	94	113	28	301	169	0.50	0.36
Li et al. (24)	26	56	22	108	100	24	37	13	85	63	0.85	0.43
You et al. (32)	20	28	12	68	52	11	15	4	37	23	0.75	0.38
Kawamoto et al. (22)	17	15	6	49	27	91	110	40	292	190	0.49	0.39
Shen et al. (29)	156	245	112	557	469	610	825	397	2,045	1,619	0.00	0.44
Mao et al. (26)	38	110	11	186	132	82	100	16	264	132	0.06	0.33
Feng et al. (21)	123	195	97	441	389	410	585	268	1,405	1,121	0.03	0.44
Yoo et al. (19)	125(CC+CT)		36(TT)			553(CC+CT)		105(TT)				

HWE, Hardy–Weinberg equilibrium; MAF, minor allele frequency; C, cytosine; T, thymine; CC, Homozygous wild-type; CT, heterozygous; TT, homozygous mutant; CC+CT, individuals carrying at least one C allele (heterozygous CT or homozygous CC).

### 3.3 Association between the MTHFR C677T gene polymorphism and CSVD

**Figure 2:**
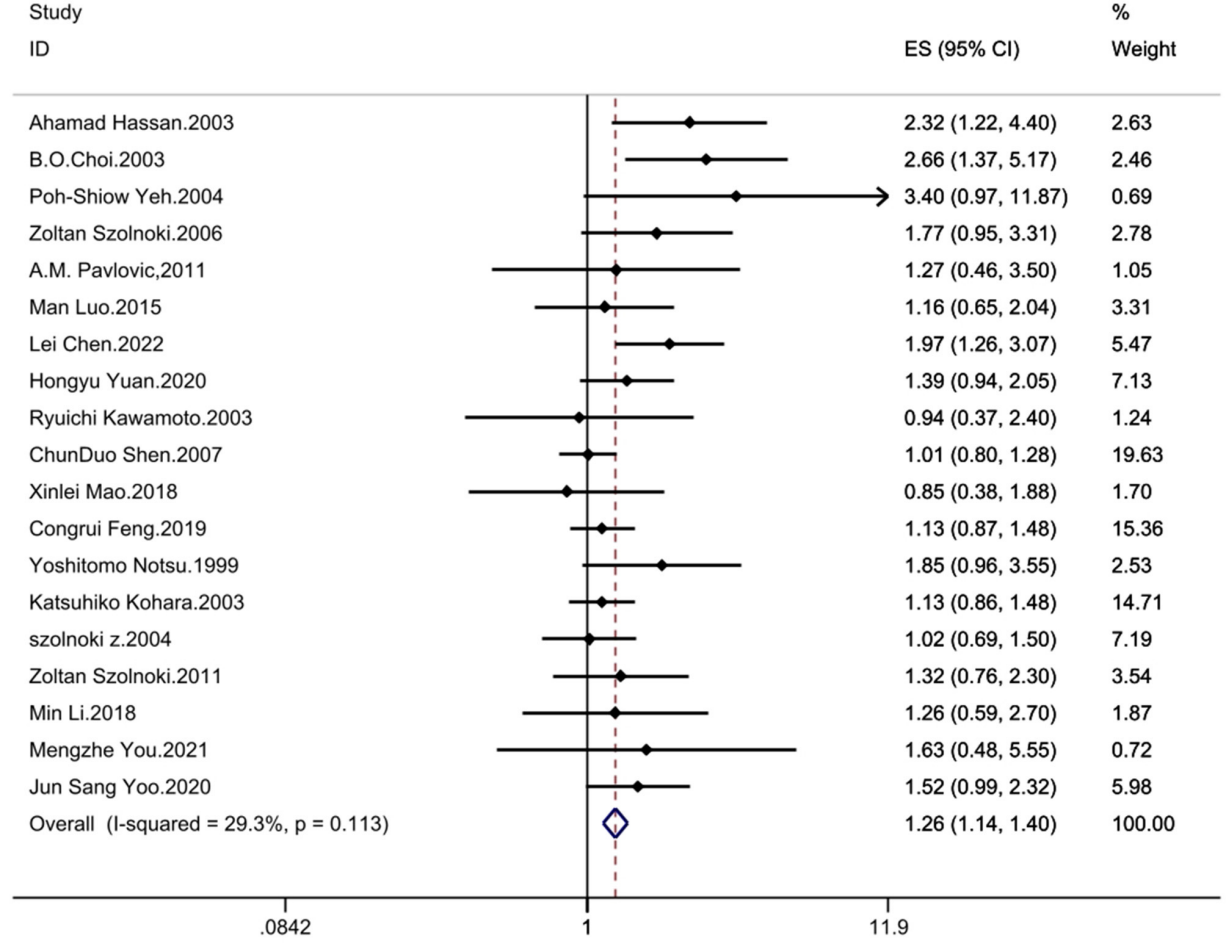
Forest plot of MTHFR C677T and CSVD (recessive model: TT vs. CT + CC).

**Figure 3:**
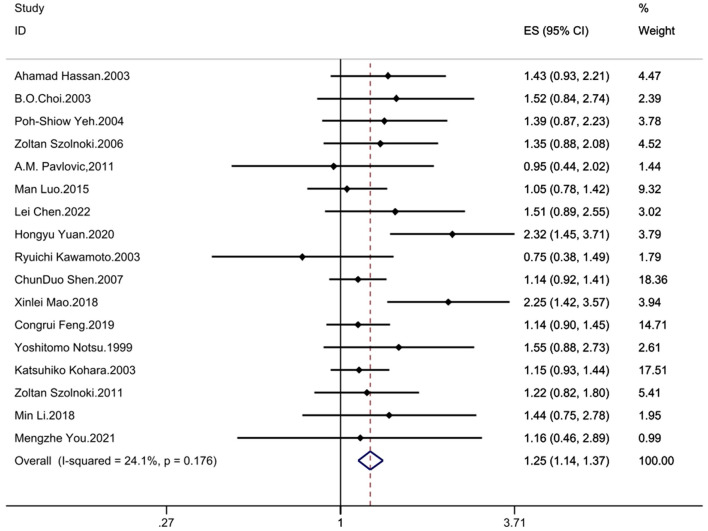
Forest plot of MTHFR C677T and CSVD (dominant model: TT + CT vs. CC).

**Figure 4:**
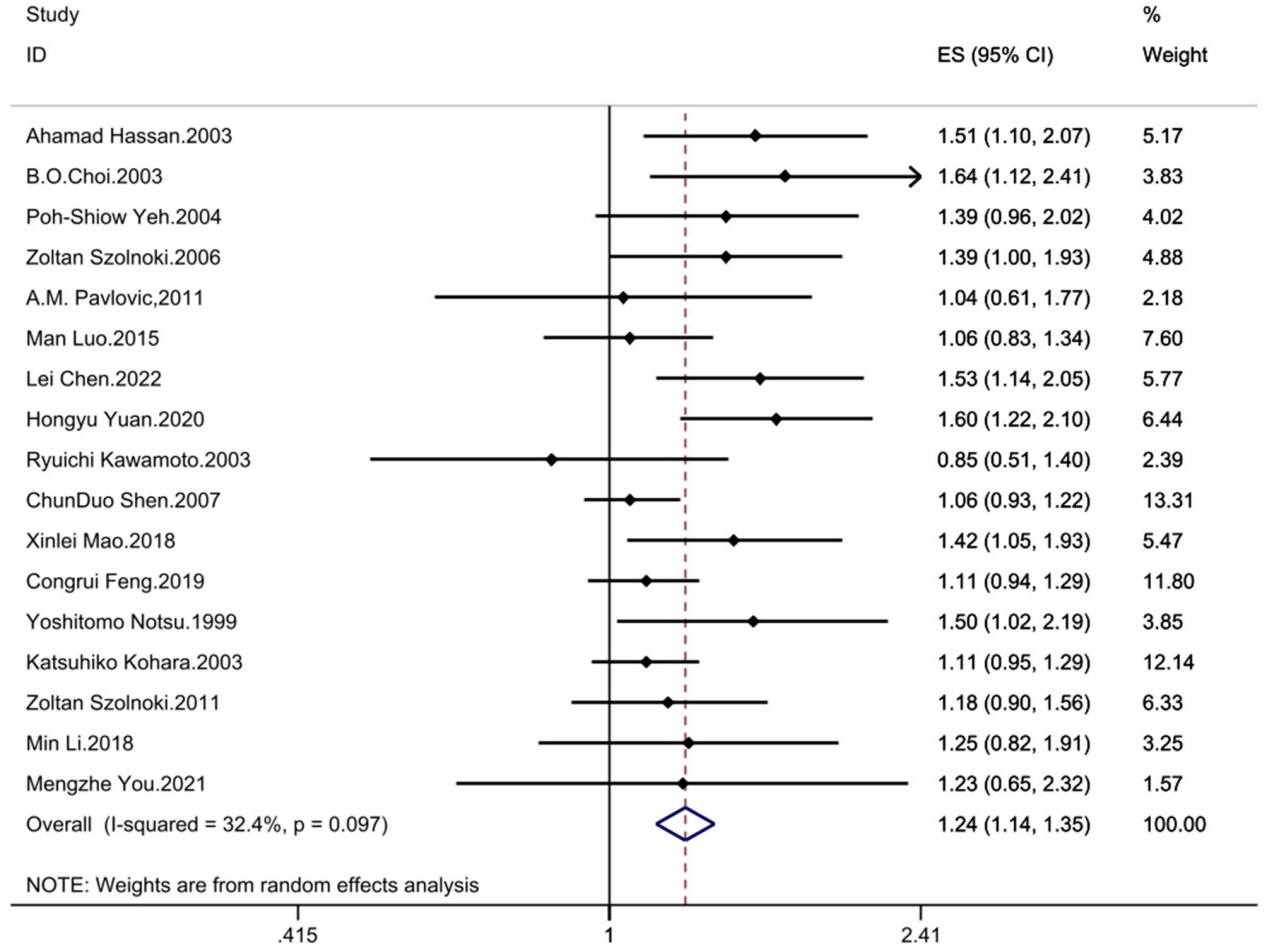
Forest plot of MTHFR C677T and CSVD (allelic model: T vs. C).

**Figure 5:**
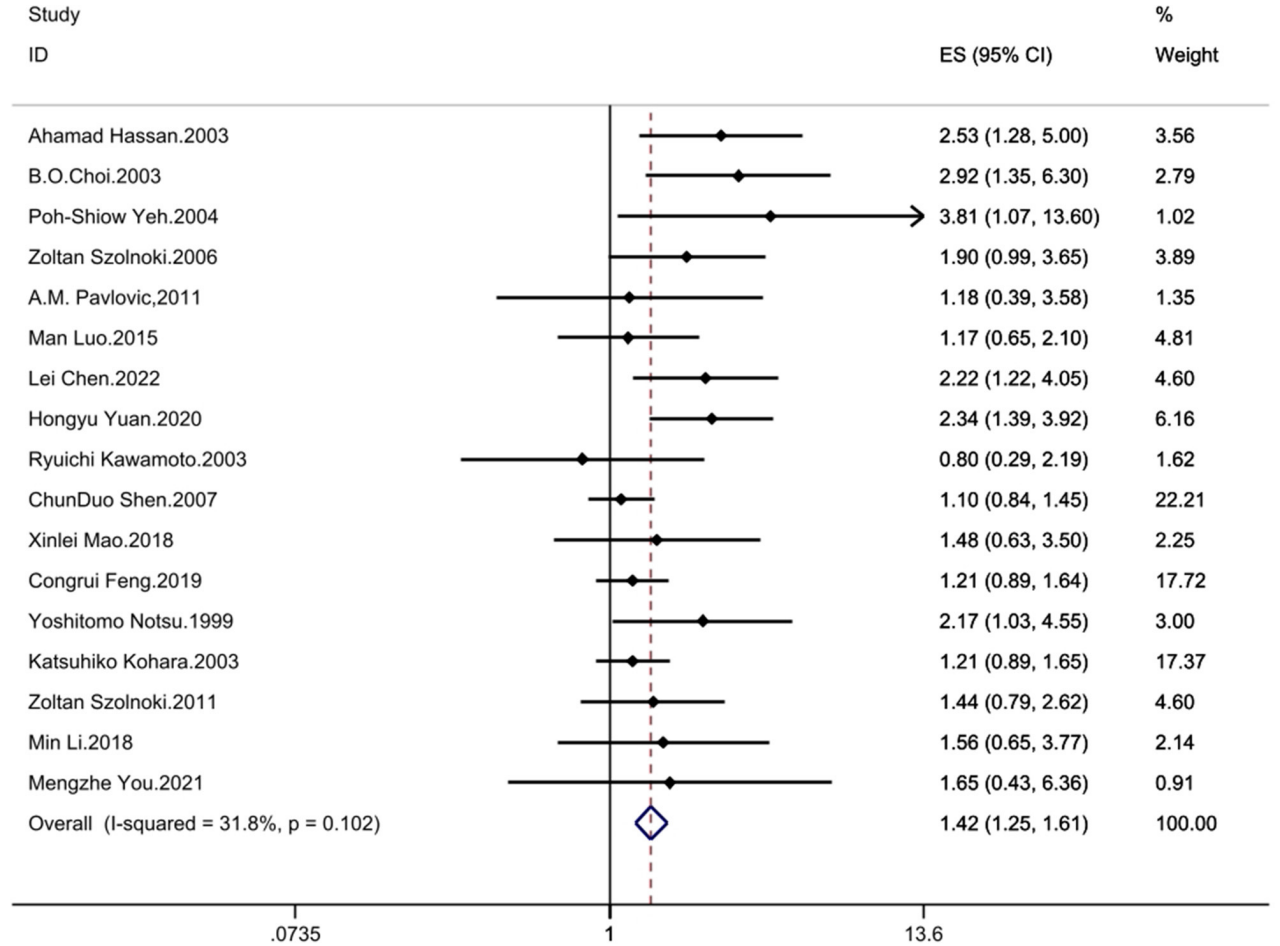
Forest plot of MTHFR C677T and CSVD (homozygote contrast: TT vs. CC).

**Figure 6:**
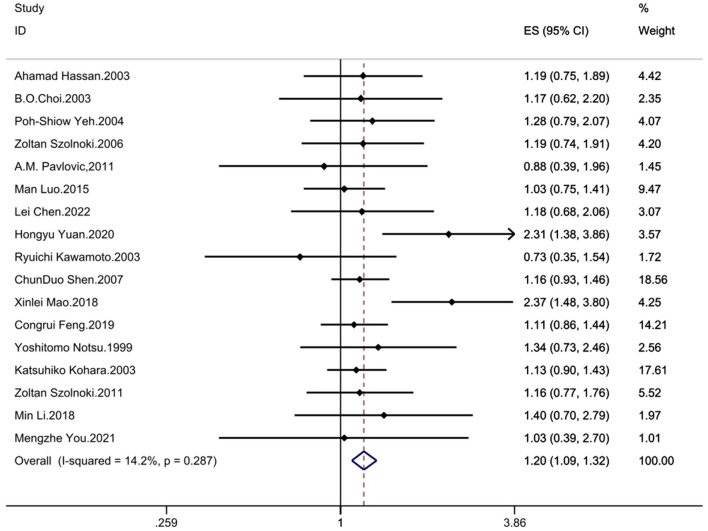
Forest plot of MTHFR C677T and CSVD (heterozygote genotype vs. wild homozygote genotype contrast: CT vs. CC).

The results demonstrated significant associations between the MTHFR C677T polymorphism and CSVD across all genetic models: recessive model (OR = 1.33; 95%CI = 1.16, 1.52; fixed effects), dominant model (OR = 1.25; 95%CI = 1.14, 1.37; fixed effects), allelic model (OR = 1.24; 95%CI= 1.14, 1.35; random effects), homozygote contrast (TT vs. CC: OR = 1.42; 95%CI = 1.25, 1.61; fixed effects), and heterozygote contrast (CT vs. CC: OR = 1.20; 95%CI = 1.09, 1.32; fixed effects) (Table 3, Figures 2–6).

**Table 3:** Main results of the pooled ORs in meta-analysis of the C677T polymorphism.

						**Test of publication bias**					
	**Sample size**	**Test of association**		**Test of heterogeneity**		**Begg's**		**Egger's**		**Result after trim-and-fill method**	
**Genetic model**	**Case/control**	**OR**	**95%CI**	***I**^2^* **(%)**	**P**	**z**	**p**	**t**	**p**	**OR**	**95%CI**
TT vs. TC+CC	3,676/8,765	1.26	1.14–1.40	29.3	0.113	2.87	0.004	4.48	0.000	1.10	0.81–1.49
TT + TC vs. CC	3,200/7,461	1.25	1.14–1.37	24.1	0.176	2.76	0.006	2.65	0.018	1.12	0.85–1.49
T vs. C	3,200/7,461	1.24	1.14–1.35	32.4	0.097	2.27	0.023	3.59	0.003	1.10	0.90–1.33
TT vs. CC	3,200/7,461	1.42	1.25–1.61	31.8	0.102	2.76	0.006	4.19	0.001	1.18	0.79–1.76
CT vs. CC	3,200/7,461	1.20	1.09–1.32	14.2	0.287	1.94	0.053	1.64	0.123	1.09	0.82–1.45

TT vs. CT + CC, recessive model; TT + CT vs. CC, dominant model; T vs. C, allelic model; TT vs. CC, homozygote contrast; CT vs. CC, heterozygote genotype vs. wild homozygote genotype contrast; OR, odd ratio; CI, confidence interval.

### 3.4 Subgroup analysis and meta-regression

Meta-regression identified disease subtype as a significant contributor to heterogeneity in the recessive model (β= −0.20; 95% CI = −0.34, −0.06; *P* = 0.009), and homozygote contrast model (TT vs. CC: β= −0.20; 95% CI= −0.37, −0.03; *P* = 0.027). No significant heterogeneity sources were identified in other subgroups (Table 4).

**Table 4:** Meta-regression of MTHFR C677T polymorphism and CSVD subgroup.

**Subgroup**	**Genetic model**	**β**	**95%CI**	**SE**	**t**	***P***
Disease subgroup	TT vs. TC+CC	−0.20	−0.34 to -0.06	0.06	−3.07	0.009
	TT+TC vs. CC	−0.05	−0.20 to 0.11	0.07	−0.64	0.54
	T vs. C	−0.09	−0.18 to 0.00	0.40	−2.18	0.050
	TT vs. CC	−0.20	−0.37 to -0.03	0.08	−2.52	0.027
	CT vs. CC	0.01	−0.15 to 0.17	0.07	0.17	0.867
Ethnicity	TT vs. TC+CC	−0.12	−0.45 to 0.21	0.15	−0.80	0.439
	TT+TC vs. CC	0.02	−0.33 to 0.38	0.16	0.14	0.892
	T vs. C	−0.05	−0.27 to 0.16	0.10	−0.97	0.351
	TT vs. CC	−0.16	−0.59 to 0.27	0.19	−0.83	0.425
	CT vs. CC	0.06	−0.30 to 0.42	0.07	0.17	0.867
Imaging criteria	TT vs. TC+CC	−0.36	−0.75 to 0.04	0.18	−1.95	0.071
	TT+TC vs. CC	0.04	−0.39 to 0.47	0.20	0.20	0.848
	T vs. C	−0.11	−0.36 to 0.14	0.12	−0.97	0.351
	TT vs. CC	−0.27	−0.78 to 0.23	0.23	−1.17	0.264
	CT vs. CC	0.19	−0.52 to 0.15	0.15	0.95	0.360
HWE compliance	TT vs. TC+CC	−0.14	−0.44 to 0.15	0.14	−1.04	0.314
	TT+TC vs. CC	−0.19	−0.53 to 0.15	0.15	−1.24	0.240
	T vs. C	−0.12	−0.31 to 0.07	0.08	−1.39	0.190
	TT vs. CC	−0.24	−0.62 to 0.15	0.18	−1.34	0.204
	CT vs. CC	−0.19	−0.52 to 0.15	0.15	−1.22	0.247

HWE, Hardy–Weinberg equilibrium; TT vs. CT + CC, recessive model; TT + CT vs. CC, dominant model; T vs. C, allelic model; TT vs. CC, homozygote contrast; CT vs. CC, heterozygote genotype vs. wild homozygote genotype contrast.

In disease subgroup analyses, notable associations between the MTHFR C677T polymorphism and CSVD-NOS emerged across all genetic models: recessive (OR= 1.73; 95% CI = 1.40, 2.14), dominant (OR = 1.38; 95% CI = 1.15, 1.67), allelic (OR = 1.38; 95% CI= 1.22, 1.57), homozygote contrast (TT vs. CC: OR = 2.04; 95% CI = 1.60, 2.60), and heterozygote contrast (CT vs. CC: OR = 1.23; 95% CI = 1.02, 1.47). For lacunar infarction, no significant associations were observed in most genetic models. White matter hyperintensity exhibited significant associations in the dominant (OR = 1.21; 95% CI = 1.02, 1.44), allelic (OR = 1.17; 95% CI = 1.04, 1.32), and homozygote contrast (TT vs. CC: OR = 1.37; 95% CI = 1.07, 1.74). Cerebral microbleed analyses were underpowered due to limited data, though a non-significant trend emerged in the recessive model (OR = 1.52; 95% CI = 0.99, 2.33) (Table 5, Supplementary Figures 1–5).

**Table 5:** Pooled ORs of C677T polymorphism meta-analysis: subgroup-stratified main results.

**Subgroup**	**Genetic model**	**Test of association**		**Test of heterogeneity**	
		**OR**	**95%CI**	***I**^2^* **(%)**	***P***
CSVD-NOS	TT vs. TC+CC	1.73	1.40–2.14	4.2	0.398
	TT+TC vs. CC	1.38	1.15–1.67	22.5	0.250
	T vs. C	1.38	1.22–1.57	17.2	0.294
	TT vs. CC	2.04	1.60–2.60	0.0	0.437
	CT vs. CC	1.23	1.02–1.47	9.6	0.356
LI	TT vs. TC+CC	1.05	0.88–1.24	0.0	0.869
	TT+TC vs. CC	1.24	0.92–1.68	68.0	0.025
	T vs. C	1.11	0.98–1.25	25.1	0.261
	TT vs. CC	1.15	0.95–1.39	0.0	0.793
	CT vs. CC	1.26	0.90–1.75	70.5	0.017
WMH	TT vs. TC+CC	1.19	0.98–1.43	0.0	0.715
	TT+TC vs. CC	1.21	1.02–1.44	0.0	0.874
	T vs. C	1.17	1.04–1.32	0.0	0.699
	TT vs. CC	1.37	1.07–1.74	0.0	0.685
	CT vs. CC	1.17	0.97–1.40	0.0	0.963
MI	TT vs. TC+CC	1.52	0.99–2.33	-	-
EUR	TT vs. TC+CC	1.41	1.03–1.91	27.1	0.241
	TT+TC vs. CC	1.28	1.02–1.62	0.0	0.812
	T vs. C	1.30	1.10–1.54	0.0	0.538
	TT vs. CC	1.78	1.25–2.52	0.0	0.558
	CT vs. CC	1.15	0.90–1.47	0.0	0.927
ASN	TT vs. TC+CC	1.32	1.12–1.54	33.8	0.104
	TT+TC vs. CC	1.31	1.13–1.51	40.2	0.066
	T vs. C	1.24	1.12–1.36	41.2	0.060
	TT vs. CC	1.49	1.22–1.83	38.6	0.076
	CT vs. CC	1.24	1.08–1.44	33.4	0.115
MRI	TT vs. TC+CC	1.41	1.17–1.71	23.4	0.228
	TT+TC vs. CC	1.24	1.07–1.45	0.0	0.908
	T vs. C	1.25	1.12–1.40	7.0	0.376
	TT vs. CC	1.57	1.24–1.97	6.9	0.377
	CT vs. CC	1.16	0.98–1.36	0.0	0.994
CT/MRI	TT vs. TC+CC	1.25	1.03–1.53	33.2	0.152
	TT+TC vs. CC	1.33	1.11–1.60	56.3	0.019
	T vs. C	1.23	1.09–1.39	48.9	0.048
	TT vs. CC	1.49	1.15–1.93	47.2	0.056
	CT vs. CC	1.28	1.05–1.55	56.3	0.028
HWE compliance (yes)	TT vs. TC+CC	1.44	1.22–1.70	15.7	0.286
	TT+TC vs. CC	1.37	1.16–1.63	39.8	0.075
	T vs. C	1.30	1.16–1.45	32.6	0.130
	TT vs. CC	1.66	1.34–2.05	22.2	0.225
	CT vs. CC	1.28	1.07–1.52	36.4	0.099
HWE compliance (no)	TT vs. TC+CC	1.13	0.94–1.36	18.1	0.296
	TT+TC vs. CC	1.17	1.02–1.35	0.0	0.857
	T vs. C	1.12	1.02–1.23	0.0	0.452
	TT vs. CC	1.29	0.99–1.66	26.0	0.248
	CT vs. CC	1.15	0.99–1.33	0.0	0.950

CSVD-NOS, Cerebral Small Vessel Disease - Not Otherwise Specified; WMH, White Matter Hyperintensity; MI, Microbleed; MRI, magnetic resonance imaging; CT, computed tomography; EUR, European; ASN, Asian; TT vs. CT + CC, recessive model; TT + CT vs. CC, dominant model; T vs. C, allelic model; TT vs. CC, homozygote contrast; CT vs. CC, heterozygote genotype vs. wild homozygote genotype contrast.

Ethnic subgroup analyses revealed significant associations in European populations for recessive (OR = 1.41; 95% CI = 1.03, 1.91), dominant (OR = 1.28; 95% CI = 1.02, 1.62), allelic (OR = 1.30; 95% CI = 1.10, 1.54), homozygote contrast (TT vs. CC: OR = 1.78; 95% CI = 1.25, 2.53) models. Asian populations revealed stronger effect sizes across all models: recessive (OR = 1.32; 95% CI = 1.12, 1.54), dominant (OR = 1.31; 95% CI = 1.13, 1.51), allelic (OR = 1.24; 95% CI = 1.12, 1.36), homozygote contrast (TT vs. CC: OR = 1.49; 95% CI = 1.22–1.83), heterozygote contrast (CT vs. CC: OR = 1.24; 95% CI = 1.08–1.44) (Table 5, Supplementary Figures 1–5).

MRI-based studies showed significant associations in recessive (OR = 1.41, 95% CI = 1.17, 1.71), dominant (OR = 1.24; 95% CI = 1.07, 1.45), allelic (OR = 1.25; 95% CI = 1.12, 1.40), and homozygote contrast (TT vs. CC: OR = 1.57; 95% CI = 1.24, 1.97) models. CT/MRI studies maintained consistent significance across all models (OR range = 1.23, 1.49) (Table 5, Supplementary Figures 1–5).

HWE-compliant studies demonstrated robust associations: recessive (OR = 1.44; 95% CI = 1.22, 1.70), dominant (OR = 1.37; 95% CI = 1.16, 1.63), allelic (OR = 1.30; 95% CI = 1.16, 1.45), homozygote contrast (OR = 1.66; 95% CI = 1.34, 2.05), and heterozygote contrast (CT vs. CC: OR = 1.28; 95% CI = 1.07, 1.52). HWE-deviant studies exhibited attenuated effects: dominant (OR = 1.17; 95% CI = 1.02, 1.35), and allelic (OR = 1.12; 95% CI= 1.02, 1.23) (Table 5, Supplementary Figures 1–5).

### 3.5 Heterogeneity evaluation

The between study heterogeneity across genetic models was systematically evaluated through *I*^2^ statistics and Q-test (Table 3). The recessive model showed low-to-moderate heterogeneity (*I*^2^ = 29.3%, *P* = 0.113), while the dominant model exhibited minimal heterogeneity (*I*^2^ = 24.1%, *P* = 0.176). Comparable heterogeneity levels were observed in the allelic (*I*^2^ = 32.4%, *P* = 0.097) and homozygote contrast (TT vs. CC: *I*^2^ = 31.8%, *P* = 0.102) models, with the heterozygote model demonstrating the lowest heterogeneity (*I*^2^ = 14.2%, *P* = 0.287).

Disease subgroup analyses revealed minimal heterogeneity for CSVD-NOS (*I*^2^ ≤ 22.5%, *P* ≥ 0.250), significant heterogeneity in LI (dominant model: *I*^2^ = 68.0%, *P* = 0.025; heterozygote model: *I*^2^ = 70.5%, *P* = 0.017), and negligible heterogeneity in WMH (*I*^2^ = 0.0%, *P* ≥ 0.685).

Ethnic subgroups displayed differential heterogeneity patterns: European populations showed moderate heterogeneity in the recessive model (*I*^2^ = 27.1%, *P* = 0.241), other genetic models exhibited low heterogeneity. While Asian populations exhibited systematically higher heterogeneity across models (*I*^2^ = 33.4–41.2%, *P* = 0.060–0.115).

Imaging subgroups demonstrated low heterogeneity in MRI-based studies (*I*^2^ ≤ 23.4%, *P* ≥ 0.228) vs. moderate-to-high heterogeneity in CT/MRI studies (*I*^2^ = 33.2–56.3%, *P* = 0.019–0.152).

HWE stratification analyses showed consistently lower heterogeneity in HWE-compliant studies (*I*^2^ = 15.7–39.8%, *P* ≥ 0.075) compared to HWE-deviant studies (*I*^2^ ≤ 26.0%, *P* ≥ 0.248).

### 3.6 Publication bias and sensitivity analysis

To assess the potential effects of small studies, both Begg's rank correlation test and Egger's linear regression analysis were used, supplemented by a visual examination of comparison-adjusted funnel plots for each outcome (Supplementary Figures 11–15). The heterozygote model (CT vs. CC) showed no statistically significant publication bias through either method (Begg's z = 1.94, *P* = 0.053; Egger's t = 1.64, *P* = 0.123). Conversely, substantial evidence of publication bias across the other four genetic models (Table 4).

The trim-and-fill method revealed significant publication bias in the meta-analysis of the MTHFR C677T polymorphism and CSVD. After adjustment, ORs decreased to non-significant levels (recessive model: OR = 1.10, 95%CI = 0.81–1.49; dominant model: OR = 1.12, 95%CI = 0.85–1.49; allelic model: OR = 1.10, 95%CI = 0.90–1.33; TT vs. CC: OR = 1.18, 95%CI = 0.79–1.76; CT vs. CC: OR = 1.09, 95%CI = 0.82–1.45), indicating publication bias likely inflated initial estimates.

For sensitivity analysis, we employed a sequential exclusion method for each study. Upon excluding any single study, the aggregated results of the remaining studies demonstrated no significant deviation from the original combined results indicating robust stability in our findings (Supplementary Figures 6–10).

## 4 Discussion

Earlier research endeavors have begun to delineate a potential link between the MTHFR C677T polymorphism and various CSVD phenotypes, including white matter hyperintensities, lacunar infarcts, and cerebral microbleeds, conclusive evidence has been elusive. This systematic review and meta-analysis of 20 case-control studies investigates the association between MTHFR C677T polymorphism and CSVD subtypes in humans. The design of case-control studies provided our analysis with a robust capacity for causal inference. An average bias risk score of 7.1 indicates the high methodological quality of the included studies, enhancing the credibility of our findings. Key findings demonstrate significant associations across genetic models, though publication bias attenuated effects after trim-and-fill adjustment. Sensitivity analysis using sequential exclusion confirmed the stability of pooled estimates, with no single study disproportionately influencing the results.

The etiology of CSVD encompasses a multifaceted array of mechanisms, such as endothelial dysfunction, compromised blood-brain barrier integrity, oxidative stress, mitochondrial impairment, and inflammatory processes. These factors contribute to the development of arteriolar sclerosis, the tortuous course of deep white matter microvessels, and a reduction in capillary density, culminating in a cascade of pathological brain tissue alterations (35, 36). These alterations manifest as white matter injury, lacunar infarction, cerebral microbleeds, and expanded perivascular spaces. Previous studies have demonstrated that small cerebral arteries exhibit heightened sensitivity to HHcy, suggesting that the impact of HHcy on CSVD may be particularly pronounced. A significant correlation has been established between HHcy and CSVD, with HHcy identified as an independent risk factor for CSVD (37). The MTHFR C677T polymorphism has been shown to diminish enzyme activity and thermal stability, with the enzyme activity in individuals with the CT and TT genotypes being 65% and 30% of that observed in the CC genotype, respectively. Furthermore, individuals harboring the TT genotype exhibit serum homocysteine levels that are 20% to 70% higher than those of the CC genotype (38). We propose that the MTHFR C677T polymorphism diminishes the enzymatic activity and thermal stability of the enzyme, leading to dysregulated homocysteine metabolism, potentially precipitating CSVD through multiple pathways, including endothelial dysfunction, oxidation of low-density lipoproteins, and heightened oxidative stress.

In our subgroup analysis, the MTHFR C677T genotype correlated with LI, WMH and CMBs, displaying a trend, yet failing to attain statistical significance. WMH, a prevalent subtype of CSVD previously considered a benign aspect of aging, have been reevaluated in the studies as a marker indicative of compromised cerebral and cardiovascular health. Studies have consistently demonstrated that WMH is associated with an elevated risk of stroke, cognitive impairment, depressive disorders, and mortality (39). The relationship between the MTHFR C677T polymorphism and WMH is controversial, with mixed findings from previous studies. Rutten-Jacobs' study suggests a correlation between the MTHFR C677T polymorphism and WMH volume. Another study, using Mendelian randomization, suggested a correlation between MTHFR C677T polymorphism and WMH (40–42). However, several other studies have failed to replicate these findings (24, 32, 34, 43). Our analysis suggests a positive trend but without statistical significance. The possible reason may be that WMH is influenced by various factors such as age, lipids, fasting glucose, smoking, inflammatory response, vitamin B12 levels, etc., and most of the current studies did not incorporate the additional variables, which interfered with the effect of MTHFR gene polymorphisms on WMH.

Lacunar infarction, the predominant subtype of CSVD, implicates plasma homocysteine in its pathogenesis. The potential mechanisms underlying homocysteine-induced atherosclerotic thrombosis encompass endothelial dysfunction, proliferation of vascular smooth muscle cells, and oxidation of low-density lipoproteins (44, 45). Previous researches have proposed a possible association between the MTHFR C677T polymorphism and lacunar infarction, potentially through increased susceptibility to, or interaction with, high blood pressure (26, 42). However, a subset of studies has failed to establish significant correlations (21, 29). Our meta-analysis did not demonstrate a significant correlation between lacunar infarction and MTHFR genotype, which may be attributed to the omission of homocysteine and hypertension as covariates in the original studies.

CMBs research is in its initial stages. Elevated total homocysteine levels have been observed in patients exhibiting a high burden of CMBs, implying that serum tHcy levels may exert an independent influence on the occurrence of CMBs (46). The correlation between the MTHFR C677T TT genotype and CMBs may be attributed to homocysteine's role in coagulation, leading to arteriolar and venous infarctions that result in microbleeds and the initiation of microhemorrhages (47).

Although our meta-analysis identified a nominally significant association between the MTHFR C677T polymorphism and CSVD, we recognize the need to interpret these findings within clinical practice. The association of the T allele reduced MTHFR enzyme activity, and subsequent hyperhomocysteinemia provides a plausible biological basis, particularly in folate-deficient populations. This supports the potential application of MTHFR C677T genotyping in CSVD risk stratification, especially in areas with low dietary folate intake or in populations prone to micronutrient deficiencies, such as the elderly or those with malabsorption syndromes. Clinically, identification of T allele carriers may prompt targeted interventions such as homocysteine-lowering therapy and early neuroimaging surveillance. However, current guidelines do not universally recommend MTHFR genotyping for CSVD due to inconsistent evidence. Our findings suggest that such testing may be of value in specific subgroups, such as patients with early-onset CSVD who lack traditional risk factors.

This study has some limitations. First, it suffers from the potential for publication bias noted in the meta-analysis, which was the main concern. Despite our efforts to comprehensively identify and include all studies that met the inclusion criteria, the use of funnel plots and Egger's and Begg's regression analyses indicated a possible risk of publication bias. The trim-and-fill method revealed that publication bias likely influenced the initial results, and the corrected Ors became no longer significant in all genetic models. Second, the methods used to measure white matter hyperintensities varied significantly across studies, contributing to methodological heterogeneity. Notably, many studies reported high inter-rater reliability; however, the subjective nature of assessing white matter hyperintensities using visual rating scales cannot be overlooked. The inclusion of CT and MRI studies introduced further heterogeneity. The lower sensitivity of CT in detecting lacunar infarctions and white matter hyperintensities might have led to underrepresentation in such studies. However, sensitivity analyses revealed that the exclusion of these studies did not significantly alter the pooled log OR values. Consequently, we deemed their inclusion to be justified and appropriate.

## 5 Conclusion

This study highlights the significant association between MTHFR C677T genotyping and CSVD. Early assessment of MTHFR C677T genotyping during the clinical evaluation of elderly patients may improve patient management and reduce the adverse prognostic impact of the CSVD burden. However, further validation of these findings in large-scale, high-quality prospective studies is required.

## Data Availability

The datasets presented in this study can be found in online repositories. The names of the repository/repositories and accession number(s) can be found in the article/Supplementary material.
