# Supplementary material for: Association between methylenetetrahydrofolate reductase C677T polymorphism and cerebral small vessel disease: a systematic review and meta-analysis
Source: Front Neurol. 2025 Mar 20;16:1556535. doi: 10.3389/fneur.2025.1556535 (PMC11965132; doi:10.3389/fneur.2025.1556535)
Supplement: Supplementary file 1 [file Supplementary_file_1.pdf]

Table S1: PRISMA 2020 Checklist

Section and Topic	Item #	Checklist item	Location where item is reported
**TITLE**			
Title	1	Identify the report as a systematic review.	Pg. 1
**ABSTRACT**			
Abstract	2	See the PRISMA 2020 for Abstracts checklist.	Pg. 1
**INTRODUCTION**			
Rationale	3	Describe the rationale for the review in the context of existing knowledge.	Pg. 2
Objectives	4	Provide an explicit statement of the objective(s) or question(s) the review addresses.	Pg. 2
**METHODS**			
Eligibility criteria	5	Specify the inclusion and exclusion criteria for the review and how studies were grouped for the syntheses.	Pg. 2
Information sources	6	Specify all databases, registers, websites, organizations, reference lists and other sources searched or consulted to identify studies. Specify the date when each source was last searched or consulted.	Pg. 2
Search strategy	7	Present the full search strategies for all databases, registers and websites, including any filters and limits used.	Supplementary
Selection process	8	Specify the methods used to decide whether a study met the inclusion criteria of the review, including how many reviewers screened each record and each report retrieved, whether they worked independently, and if applicable, details of automation tools used in the process.	Pg. 2, Figure 1
Data collection process	9	Specify the methods used to collect data from reports, including how many reviewers collected data from each report, whether they worked independently, any processes for obtaining or confirming data from study investigators, and if applicable, details of automation tools used in the process.	Pg. 3
Data items	10a	List and define all outcomes for which data were sought. Specify whether all results that were compatible with each outcome domain in each study were sought (e.g. for all measures, time points, analyses), and if not, the methods used to decide which results to collect.	Pg. 5-6
	10b	List and define all other variables for which data were sought (e.g. participant and intervention characteristics, funding sources). Describe any assumptions made about any missing or unclear information.	Table1
Study risk of bias assessment	11	Specify the methods used to assess risk of bias in the included studies, including details of the tool(s) used, how many reviewers assessed each study and whether they worked independently, and if applicable, details of automation tools used in the process.	Pg. 3
Effect measures	12	Specify for each outcome the effect measure(s) (e.g. risk ratio, mean difference) used in the synthesis or presentation of results.	Pg. 6

Synthesis methods	13a	Describe the processes used to decide which studies were eligible for each synthesis (e.g. tabulating the study intervention characteristics and comparing against the planned groups for each synthesis (item #5)).	Table 1
	13b	Describe any methods required to prepare the data for presentation or synthesis, such as handling of missing summary statistics, or data conversions.	Pg. 6
	13c	Describe any methods used to tabulate or visually display results of individual studies and syntheses.	Pg. 6
	13d	Describe any methods used to synthesize results and provide a rationale for the choice(s). If meta-analysis was performed, describe the model(s), method(s) to identify the presence and extent of statistical heterogeneity, and software package(s) used.	Pg. 6
	13e	Describe any methods used to explore possible causes of heterogeneity among study results (e.g. subgroup analysis, meta-regression).	Pg. 6
	13f	Describe any sensitivity analyses conducted to assess robustness of the synthesized results.	Pg. 6
Reporting bias assessment	14	Describe any methods used to assess risk of bias due to missing results in a synt6esis (arising from reporting biases).	Pg. 6

**Section and Topic**	**Item #**	**Checklist item**	**Location where item is reported**
Certainty assessment	15	Describe any methods used to assess certainty (or confidence) in the body of evidence for an outcome.	None
**RESULTS**			
Study selection	16a	Describe the results of the search and selection process, from the number of records identified in the search to the number of studies included in the review, ideally using a flow diagram.	Figure 1
	16b	Cite studies that might appear to meet the inclusion criteria, but which were excluded, and explain why they were excluded.	None
Study characteristics	17	Cite each included study and present its characteristics.	Table 1
Risk of bias in studies	18	Present assessments of risk of bias for each included study.	Table 1
Results of individual studies	19	For all outcomes, present, for each study: (a) summary statistics for each group (where appropriate) and (b) an effect estimate and its precision (e.g. confidence/credible interval), ideally using structured tables or plots.	Pg. 7-8, Table 3
Results of syntheses	20a	For each synthesis, briefly summarise the characteristics and risk of bias among contributing studies.	Table 3-5
	20b	Present results of all statistical syntheses conducted. If meta-analysis was done, present for each the summary estimate and its precision (e.g. confidence/credible interval) and measures of statistical heterogeneity. If comparing groups, describe the direction of the effect.	Table 4
	20c	Present results of all investigations of possible causes of heterogeneity among study results.	Table 5
	20d	Present results of all sensitivity analyses conducted to assess the robustness of the synthesized results.	Pg. 9-10,

			Supplementary Figures 6-10
Reporting biases	21	Present assessments of risk of bias due to missing results (arising from reporting biases) for each synthesis assessed.	None
Certainty of evidence	22	Present assessments of certainty (or confidence) in the body of evidence for each outcome assessed.	None
**DISCUSSION**			
Discussion	23a	Provide a general interpretation of the results in the context of other evidence.	Pg. 10-12
	23b	Discuss any limitations of the evidence included in the review.	Pg. 12
	23c	Discuss any limitations of the review processes used.	Pg. 12
	23d	Discuss implications of the results for practice, policy, and future research.	Pg. 12
**OTHER INFORMATION**			
Registration and protocol	24a	Provide registration information for the review, including register name and registration number, or state that the review was not registered.	Pg. 2
	24b	Indicate where the review protocol can be accessed, or state that a protocol was not prepared.	Pg. 2
	24c	Describe and explain any amendments to information provided at registration or in the protocol.	None
Support	25	Describe sources of financial or non-financial support for the review, and the role of the funders or sponsors in the review.	Pg. 14
Competing interests	26	Declare any competing interests of review authors.	Pg. 14
Availability of data, code and other materials	27	Report which of the following are publicly available and where they can be found: template data collection forms; data extracted from included studies; data used for all analyses; analytic code; any other materials used in the review.	Supplementary

From: Page MJ, McKenzie JE, Bossuyt PM, et al. The PRISMA 2020 statement: an updated guideline for reporting systematic reviews. *BMJ* 2021;372:n71. doi:10.1136/bmj.n71

Table S2: Search equation via PubMed, EMBASE, Cochrane library

PubMed (5228)

("CADASIL"[Title/Abstract] OR "Cerebral Autosomal Dominant Arteriopathy with Subcortical Infarcts and Leukoencephalopathy"[Title/Abstract] OR "NOTCH3"[Title/Abstract] OR "HTRA1"[Title/Abstract] OR "cathepsin A-related arteriopathy with strokes and leukoencephalopathy"[Title/Abstract] OR "HtrA Serine Peptidase 1"[Title/Abstract] OR "alpha-GAL"[Title/Abstract] OR "Fabry"[Title/Abstract] OR "COL4A1"[Title/Abstract] OR "COL4A2"[Title/Abstract] OR "Type IV collagen"[Title/Abstract] OR "Forkhead box C1"[Title/Abstract] OR "FOXC1"[Title/Abstract] OR "COL4A1"[Title/Abstract] OR "CARASIL"[Title/Abstract] OR "Cerebral autosomal recessive arteriopathy with subcortical infarcts and leukoencephalopathy"[Title/Abstract] OR ("Cerebral Small Vessel Diseases"[MeSH Terms] OR "stroke, lacunar"[MeSH Terms] OR "leukoencephalopathies"[MeSH Terms] OR (("disease"[MeSH Terms] OR "disease"[All Fields] OR "diseases"[All Fields] OR "disease s"[All Fields] OR "diseased"[All Fields]) OR ("white matter"[MeSH Terms]) OR "microbleed"[Title/Abstract] OR "susceptibility weighted imaging"[Title/Abstract] OR "SWI"[Title/Abstract] OR "small artery occlusion"[Title/Abstract] OR "SAO"[Title/Abstract] OR "Cerebral Small Vessel Diseases"[Title/Abstract] OR "CSVD"[Title/Abstract] OR "infarct"[Title/Abstract] OR "infarct lacunar"[Title/Abstract] OR "white matter"[Title/Abstract])) AND ("methylenetetrahydrofolate"[Title/Abstract] OR "MTHFR"[Title/Abstract] OR ("5,10 methylenetetrahydrofolate reductase fadh2"[MeSH Terms] OR ("methylenetetrahydrofolate reductase nadph2"[MeSH Terms] OR "Methylenetetrahydrofolate reductase deficiency"[Supplementary Concept])))

Embase (2323)

('cerebral small vessel disease'/exp OR 'cerebral small vessel disease' OR 'csvd' OR 'small vessel disease'/exp OR 'small vessel disease' OR 'svd' OR 'lacunar' OR 'subcortical infarct' OR 'small artery occlusion'/exp OR 'small artery occlusion' OR 'sao' OR 'subcortical stroke'/exp OR 'subcortical stroke' OR 'microinfarct' OR 'subcortical lesion'/exp OR 'subcortical lesion' OR 'cerebrovascular disorder'/exp OR 'cerebrovascular disorder' OR 'virchow-robin space'/exp OR 'virchow-robin space' OR 'perivascular space'/exp OR 'perivascular space' OR 'leukoaraiosis'/exp OR 'leukoaraiosis' OR 'leukoencephalopathies'/exp OR 'leukoencephalopathies' OR 'leuko-araiosis'/exp OR 'leuko-araiosis' OR 'white matter'/exp OR 'white matter' OR 'wmh' OR 'microbleed'/exp OR 'microbleed' OR 'micro hemorrhage' OR 'carasil'/exp OR 'carasil' OR 'cerebral autosomal recessive arteriopathy with subcortical infarcts and leukoencephalopathy'/exp OR 'cerebral autosomal recessive arteriopathy with subcortical infarcts and leukoencephalopathy' OR htra1 OR 'cathepsin a-related arteriopathy with strokes and leukoencephalopathy' OR 'htra serine peptidase 1'/exp OR 'htra serine peptidase 1' OR 'alpha-gal' OR 'fabry' OR 'col4a2' OR 'forkhead box c1' OR 'foxc1' OR 'col4a1' OR 'brain atrophy'/exp OR 'brain atrophy')
AND ('methylenetetrahydrofolate'/exp OR methylenetetrahydrofolate OR mthfr OR 'c677t')

Cochrance (6)

(((“cerebral small vessel disease”):ti,ab,kw) OR ((“cerebral small vessel disease”):ti,ab,kw) OR ((“csvd”) :ti,ab,kw) OR ((“small vessel disease”):ti,ab,kw) OR ((“small vessel disease”) :ti,ab,kw) OR ((“svd”) :ti,ab,kw) OR ((“lacunar”) :ti,ab,kw) OR ((“subcortical infarct”) :ti,ab,kw) OR ((“small artery occlusion”):ti,ab,kw) OR ((“small artery occlusion”) :ti,ab,kw) OR ((“sao”) :ti,ab,kw) OR ((“subcortical stroke”):ti,ab,kw) OR ((“subcortical stroke”) :ti,ab,kw) OR ((“microinfarct”) :ti,ab,kw) OR ((“subcortical lesion”):ti,ab,kw) OR ((“subcortical lesion”) :ti,ab,kw) OR ((“cerebrovascular disorder”):ti,ab,kw) OR ((“cerebrovascular disorder”) :ti,ab,kw) OR ((“virchow-robin space”):ti,ab,kw) OR ((“virchow-robin space”) :ti,ab,kw) OR ((“perivascular space”):ti,ab,kw) OR ((“perivascular space”) :ti,ab,kw) OR ((“leukoaraiosis”):ti,ab,kw) OR ((“leukoaraiosis”) :ti,ab,kw) OR ((“leukoencephalopathies”):ti,ab,kw) OR ((“leukoencephalopathies”) :ti,ab,kw) OR ((“leuko-araiosis”):ti,ab,kw) OR ((“leuko-araiosis”) :ti,ab,kw) OR ((“white matter”):ti,ab,kw) OR ((“white matter”) :ti,ab,kw) OR ((“WMH”) :ti,ab,kw) OR ((“microbleed”):ti,ab,kw) OR ((“microbleed”) :ti,ab,kw) OR ((“micro hemorrhage”) :ti,ab,kw) OR ((“carasil”):ti,ab,kw) OR ((“carasil”) :ti,ab,kw) OR ((“cerebral autosomal recessive arteriopathy with subcortical infarcts and leukoencephalopathy”):ti,ab,kw) OR ((“cerebral autosomal recessive arteriopathy with subcortical infarcts and leukoencephalopathy”) :ti,ab,kw) OR ((“htra1”) :ti,ab,kw) OR ((“cathepsin a-related arteriopathy with strokes and leukoencephalopathy”) :ti,ab,kw) OR ((“htra serine peptidase 1”):ti,ab,kw) OR ((“htra serine peptidase 1”) :ti,ab,kw) OR ((“alpha-gal”) :ti,ab,kw) OR ((“fabry”) :ti,ab,kw) OR ((“col4a2”) :ti,ab,kw) OR ((“forkhead box c1”) :ti,ab,kw) OR ((“foxc1”) :ti,ab,kw) OR ((“col4a1”) :ti,ab,kw) OR ((“brain atrophy”):ti,ab,kw) OR ((“brain atrophy”) :ti,ab,kw))
AND (((“methylenetetrahydrofolate”):ti,ab,kw) OR ((“methylenetetrahydrofolate”) :ti,ab,kw) OR ((“mthfr”) :ti,ab,kw) OR ((“c677t”) :ti,ab,kw))

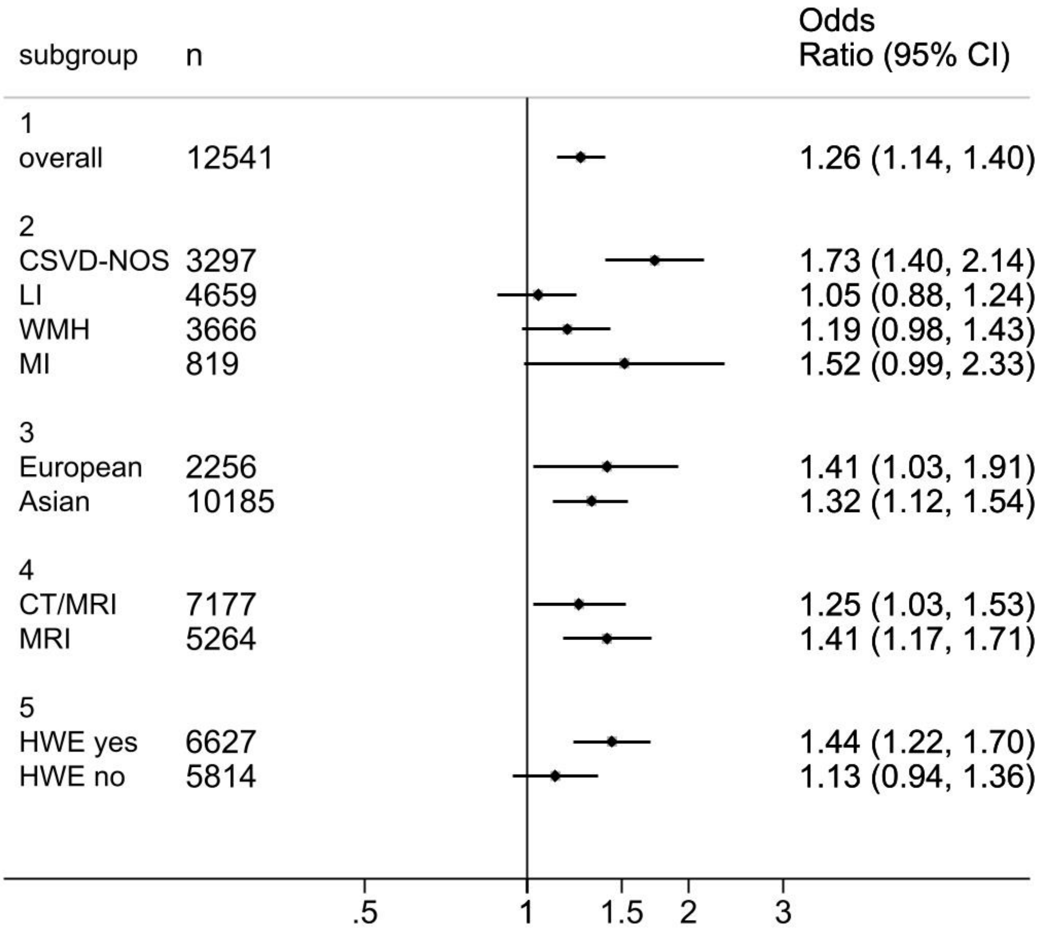

Figure 1. Forest plot of MTHFR C677T and CSVD subgroup (recessive model: TT vs. CT + CC).

Abbreviations: CSVD-NOS, Cerebral Small Vessel Disease - Not Otherwise Specified; MI, cerebral microbleeds; Li, lacunar infarction; WMH, white matter hyperintensity; HWE, Hardy − Weinberg equilibrium.

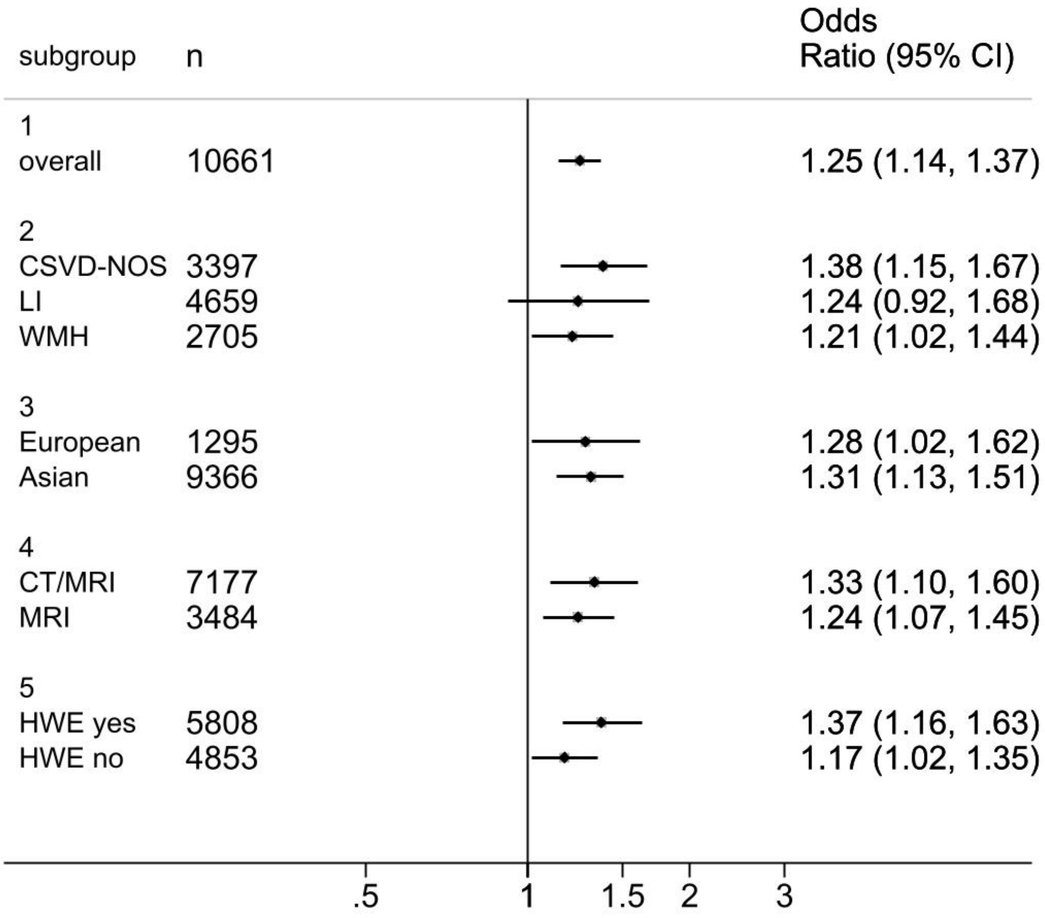

Figure 2. Forest plot of MTHFR C677T and CSVD subgroup (dominant model: TT + CT vs. CC).

Abbreviations: CSVD-NOS, Cerebral Small Vessel Disease - Not Otherwise Specified; MI, cerebral microbleeds; Li, lacunar infarction; WMH, white matter hyperintensity; HWE, Hardy − Weinberg equilibrium.

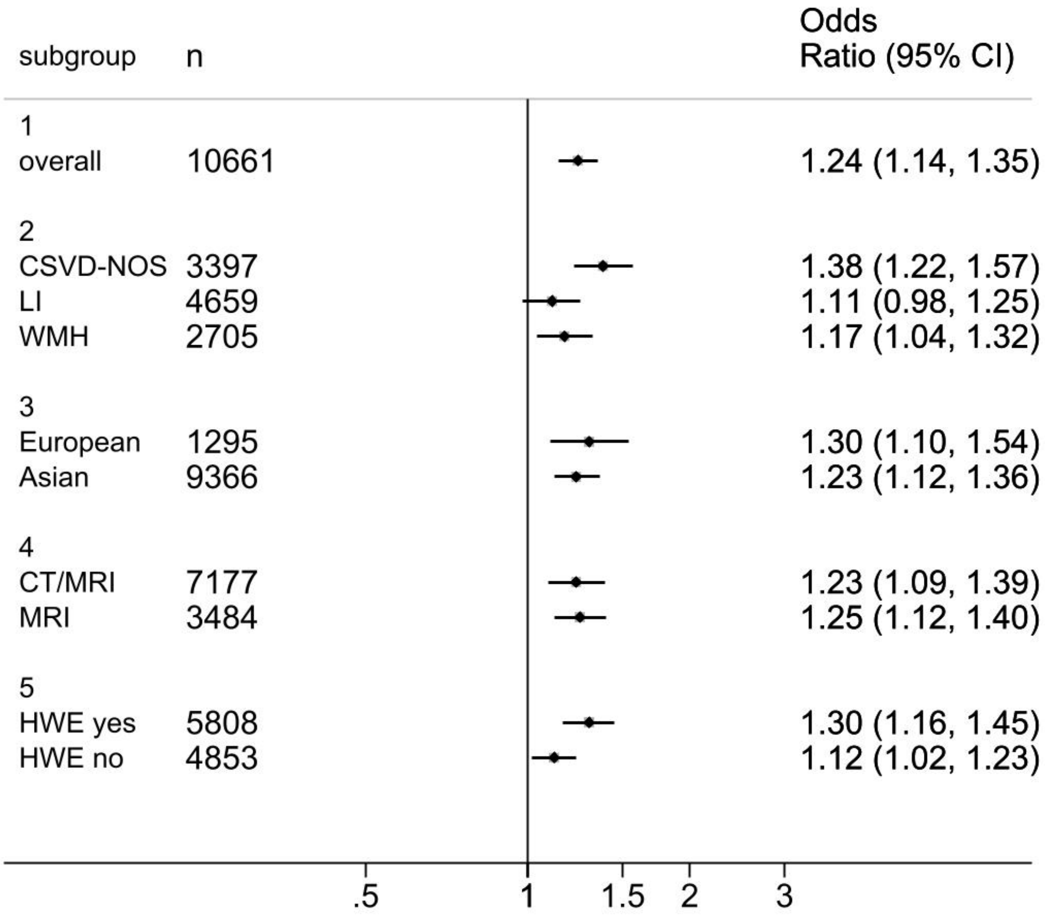

Figure 3. Forest plot of MTHFR C677T and CSVD subgroup (allelic model: T vs. C).

Abbreviations: CSVD-NOS, Cerebral Small Vessel Disease - Not Otherwise Specified; MI, cerebral microbleeds; Li, lacunar infarction; WMH, white matter hyperintensity; HWE, Hardy – Weinberg equilibrium.

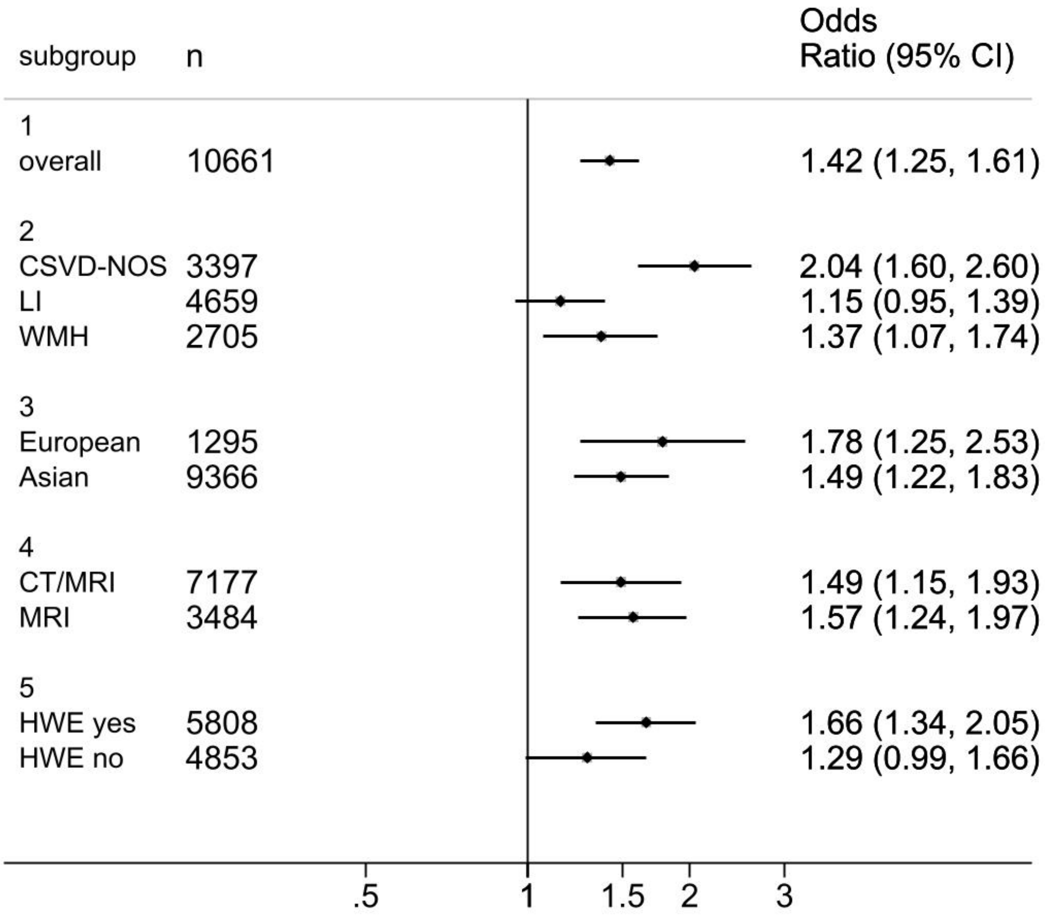

Figure 4. Forest plot of MTHFR C677T and CSVD subgroup (homozygote contrast: TT vs. CC).

Abbreviations: CSVD-NOS, Cerebral Small Vessel Disease - Not Otherwise Specified; MI, cerebral microbleeds; Li, lacunar infarction; WMH, white matter hyperintensity; HWE, Hardy – Weinberg equilibrium.

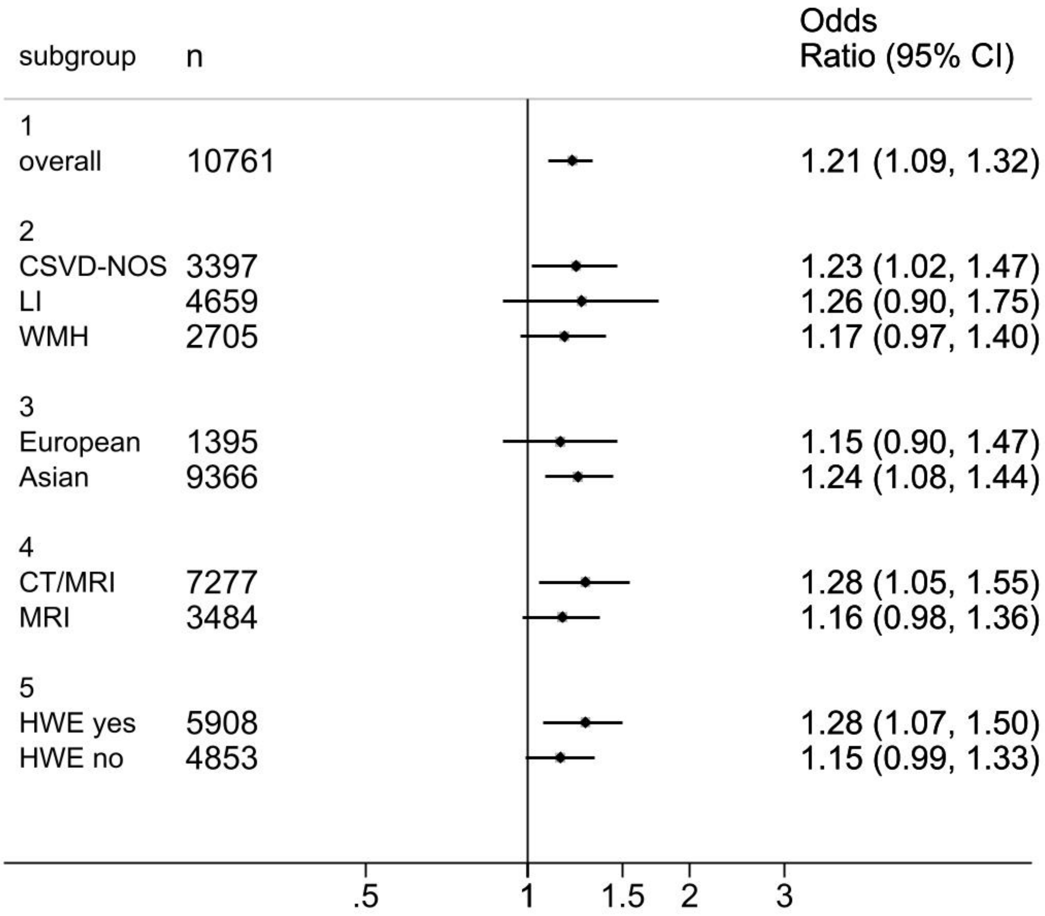

Figure 5. Forest plot of MTHFR C677T and CSVD subgroup heterozygote genotype vs. wild homozygote genotype contrast: CT vs. CC).

Abbreviations: CSVD-NOS, Cerebral Small Vessel Disease - Not Otherwise Specified; MI, cerebral microbleeds; Li, lacunar infarction; WMH, white matter hyperintensity; HWE, Hardy－Weinberg equilibrium.

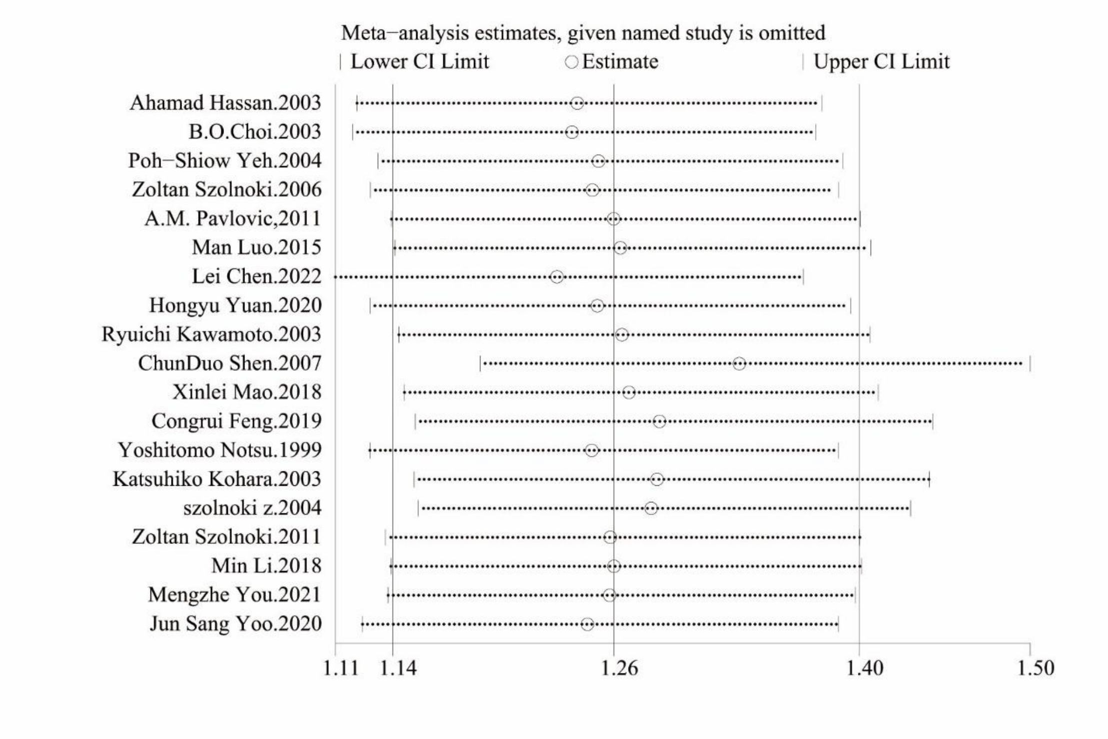

Figure 6. The sensitivity analysis of prevalence (recessive model: TT vs. CT + CC).

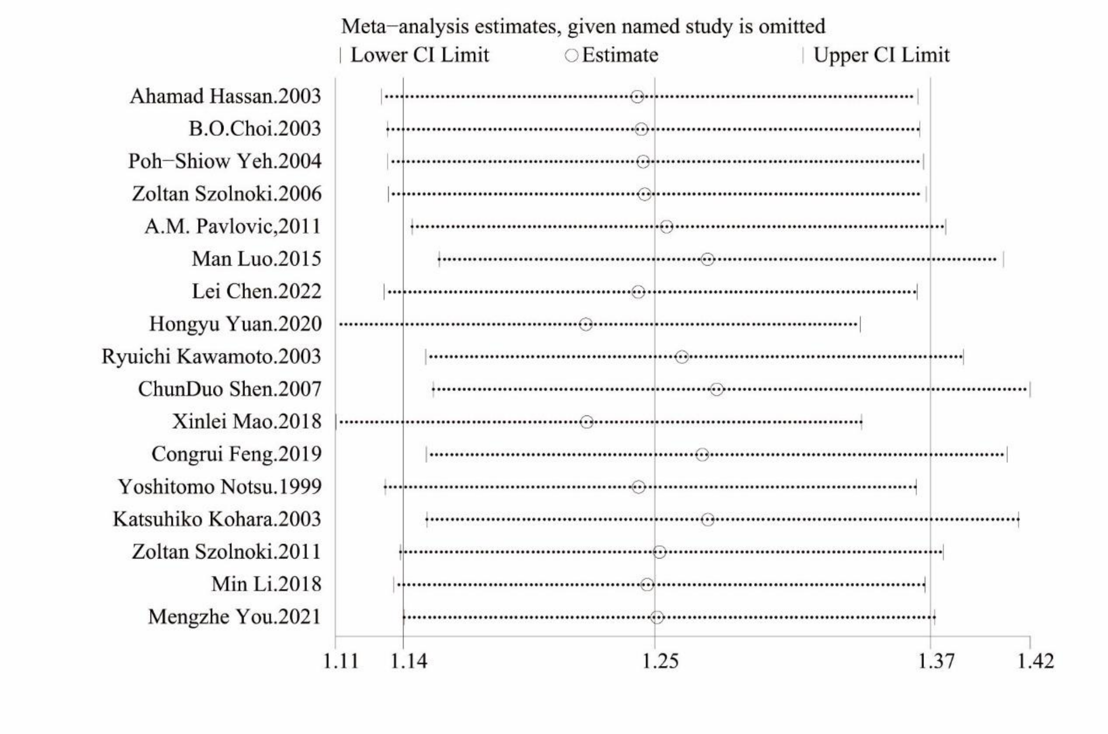

Figure 7. The sensitivity analysis of prevalence (dominant model: TT + CT vs. CC).

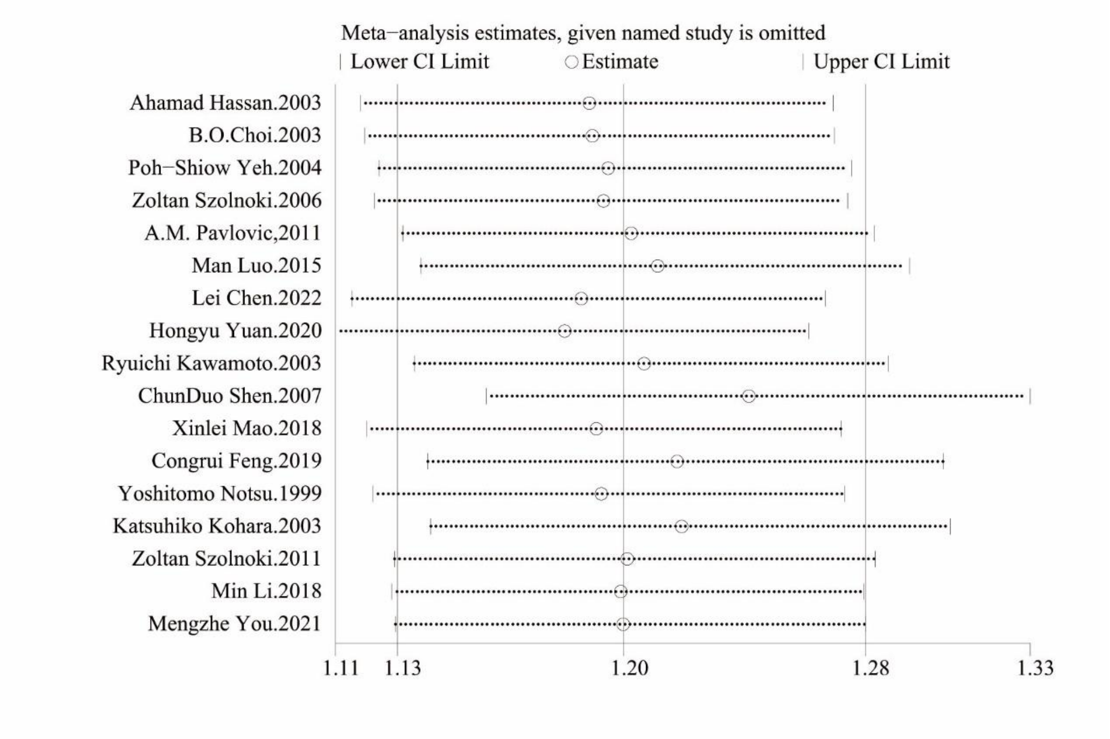

Figure 8. The sensitivity analysis of prevalence (allelic model: T vs. C).

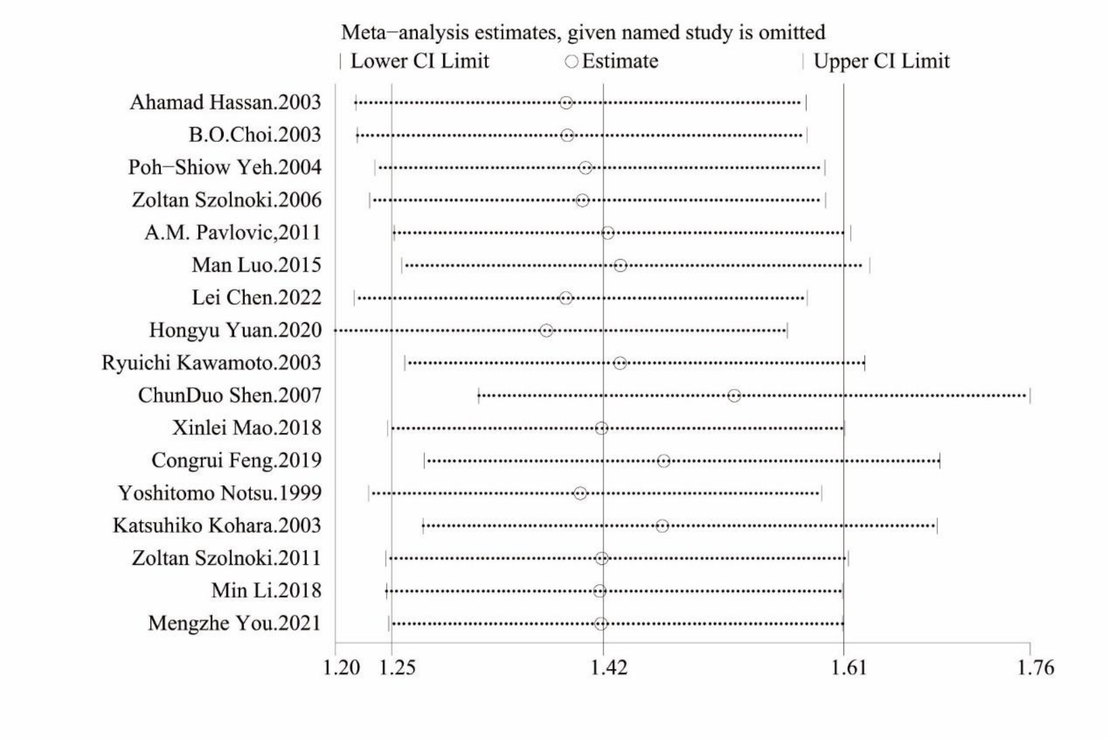

Figure 9. The sensitivity analysis of prevalence (homozygote contrast: TT vs. CC).

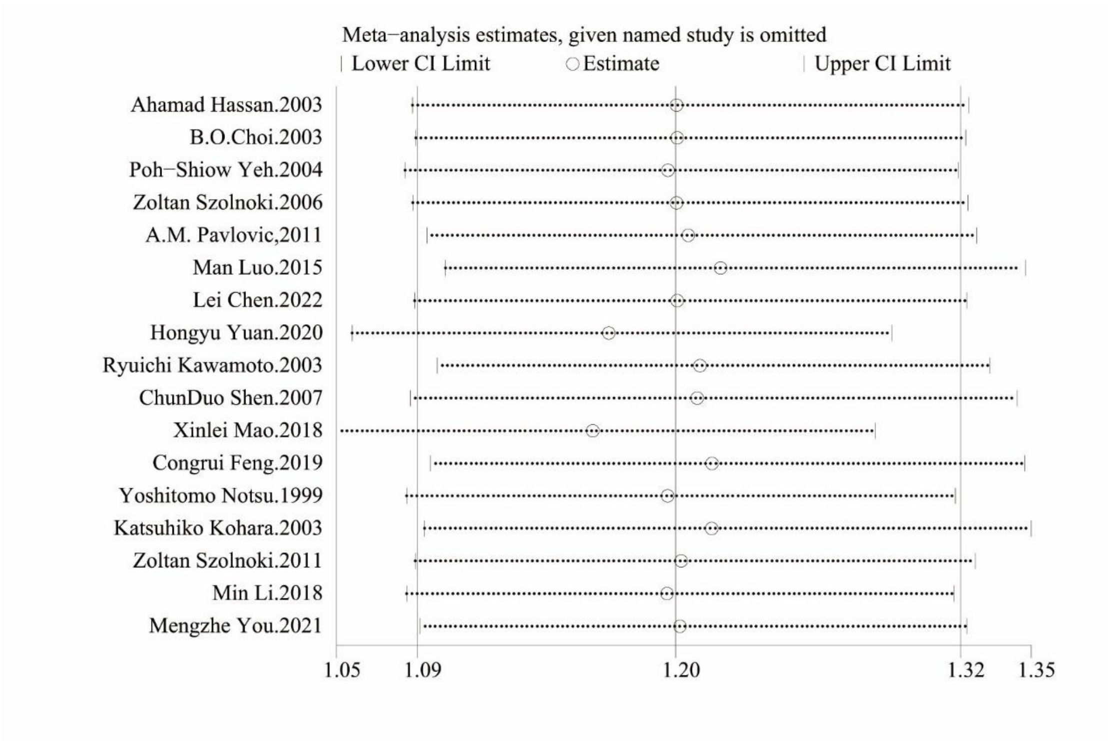

Figure 10. The sensitivity analysis of prevalence (heterozygote genotype vs. wild homozygote genotype contrast: CT vs. CC).

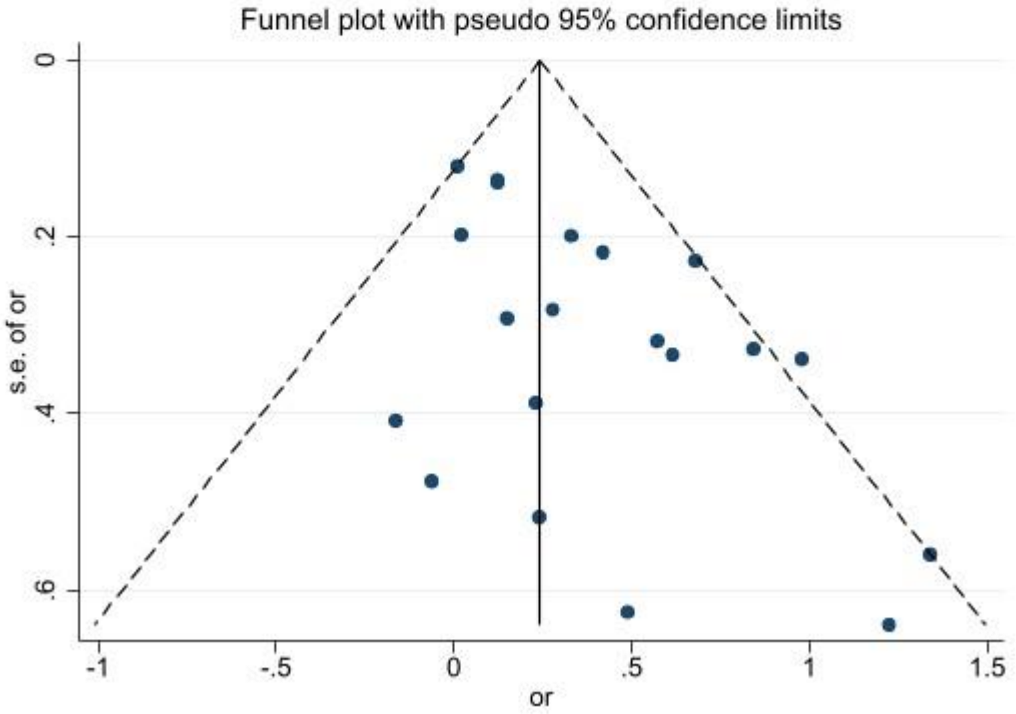

Figure 11. Funnel plot (recessive model: TT vs. CT + CC).

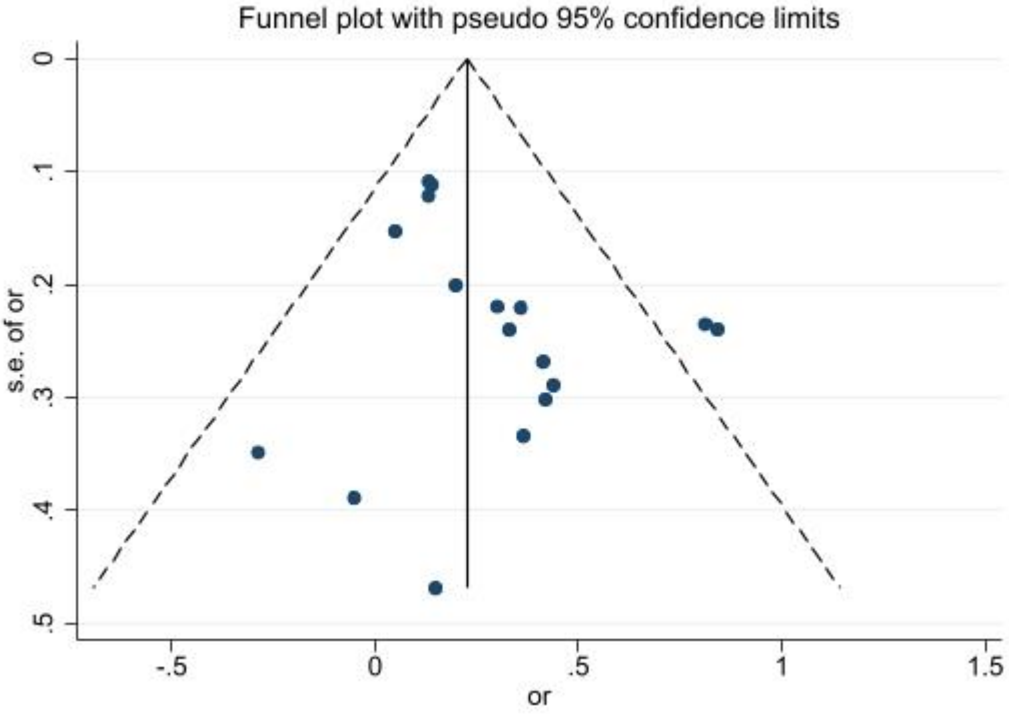

Figure 12. Funnel plot (dominant model: TT + CT vs. CC).

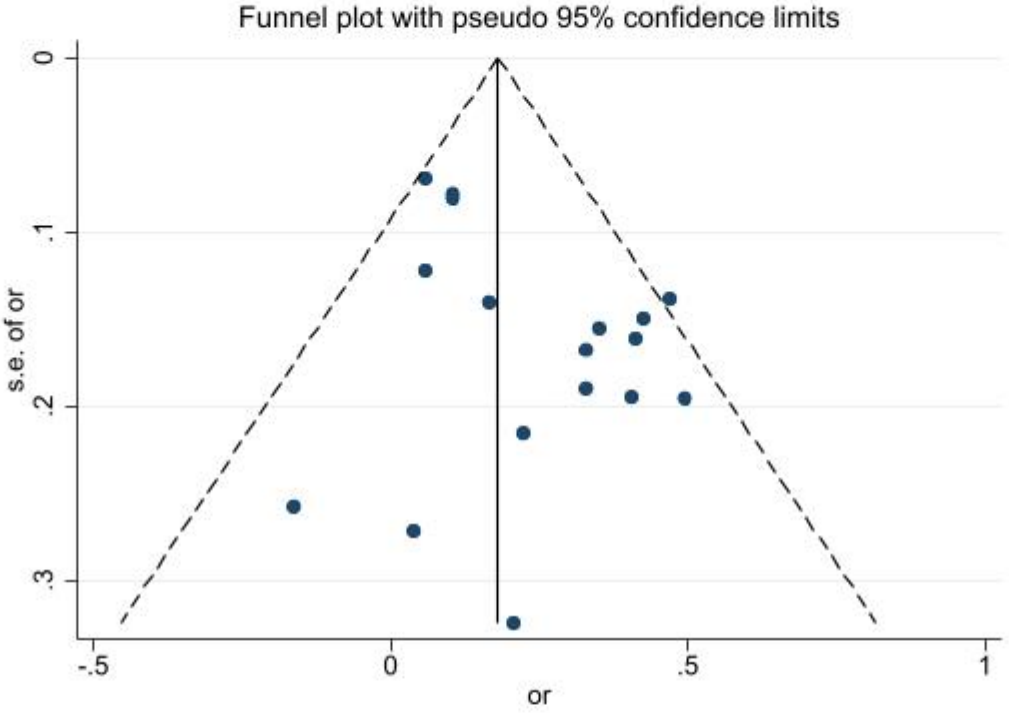

Figure 13. Funnel plot (allelic model: T vs. C).

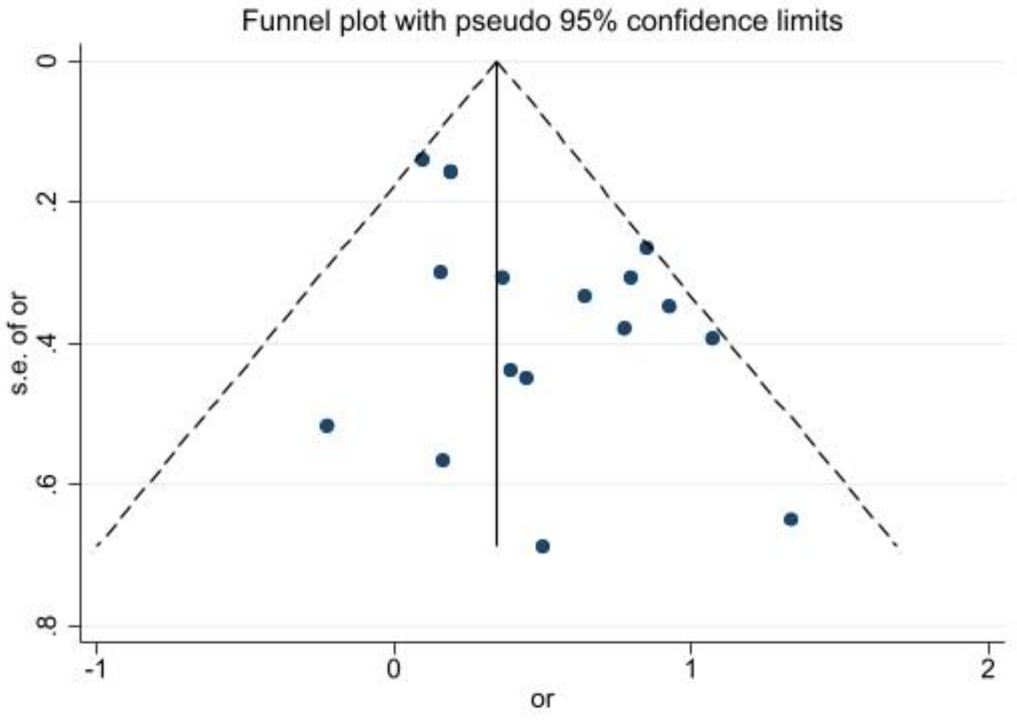

Figure 14. Funnel plot (homozygote contrast: TT vs. CC).

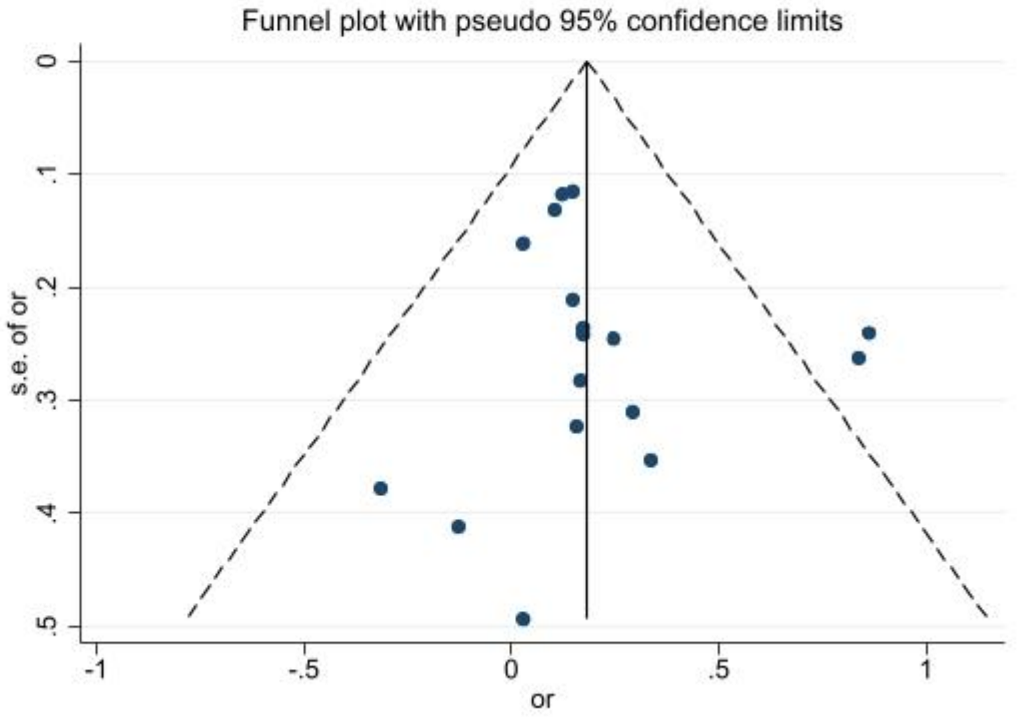

Figure 15. Funnel plot (heterozygote genotype vs. wild homozygote genotype contrast: CT vs. CC).

References

1. Duering M, Biessels GJ, Brodtmann A, Chen C, Cordonnier C, de Leeuw FE, et al. Neuroimaging standards for research into small vessel disease-advances since 2013. *Lancet Neurol.* (2023) 22:602–18. doi: 10.1016/S1474-4422(23)00131-X

2. Huo Y, Li J, Qin X, et al. Efficacy of folic acid therapy in primary prevention of stroke among adults with hypertension in China: the CSPPT randomized clinical trial. JAMA 2015;313(13):1325-35. DOI: 10.1001/jama.2015.2274.

3. Valdes HMC, Morris Z, Dickie DA, Royle NA, Muñoz Maniega S, Aribisala BS, et al. Close correlation between quantitative and qualitative assessments of white matter lesions. *Neuroepidemiology.* (2013) 40:13–22. doi: 10.1159/000341859

4. Hassan A. Homocysteine is a risk factor for cerebral small vessel disease, acting via endothelial dysfunction. *Brain.* (2004) 127:212–9. doi: 10.1093/brain/awh023

5. Yuan H, Fu M, Yang X, Huang K, Ren X. Single nucleotide polymorphism ofMTHFR rs1801133 associated with elevated Hcy levels affects susceptibility to cerebral small vessel disease. *PeerJ.* (2020) 8:e8627. doi: 10.7717/peerj.8627

6. Cao Y, Su N, Zhang D, Zhou L, Yao M, Zhang S, et al. Correlation between total homocysteine and cerebral small vessel disease: a Mendelian randomization study. *Eur J Neurol.* (2021) 28:1931–8. doi: 10.1111/ene.14708

7. Feng C, Bai X, Xu Y, Hua T, Huang J, Liu X-Y. Hyperhomocysteinemia associates with small vessel disease more closely than large vessel disease. *Int J Med Sci.* (2013) 10:408–12. doi: 10.7150/ijms.5272

8. Nam K-W, Kwon H-M, Jeong H-Y, Park J-H, Kwon H, Jeong S-M. Serum homocysteine level is related to cerebral small vessel disease in a healthy population. *Neurology.* (2019) 92:e317–25. doi: 10.1212/WNL.0000000000006816

9. Annus Á, Juhász LÁ, Szabó E, Rárosi F, Szpisjak L, Vécsei L, et al. Connection between small vessel disease related stroke and the MTHFR C677T polymorphism in a Hungarian population. *Heliyon.* (2020) 6:e05305. doi: 10.1016/j.heliyon.2020.e05305

10. Jacob MA, Cai M, van de Donk V, Bergkamp M, Marques J, Norris DG, et al. Cerebral small vessel disease progression and the risk of dementia: a 14-year follow-up study. *Am J Psychiatry.* (2023) 180:508–18. doi: 10.1176/appi.ajp.20220380

11. Mishra A, Duplaa C, Vojinovic D, Suzuki H, Sargurupremraj M, Zilhão NR, et al. Gene-mapping study of extremes of cerebral small vessel disease reveals TRIM47 as a strong candidate. *Brain.* (2022) 145:1992–2007. doi: 10.1093/brain/awab432

12. Rudilosso S, Mena L, Esteller D, Olivera M, Mengual JJ, Montull C, et al. Higher Cerebral Small Vessel Disease Burden in Patients with White Matter Recent Small Subcortical Infarcts. *J Stroke Cerebrovasc Dis.* (2021) 30:105824. doi:10.1016/j.jstrokecerebrovasdis.2021.105824

13. Sargurupremraj M, Suzuki H, Jian X, Sarnowski C, Evans TE, Bis JC, et al. Cerebral small vessel disease genomics and its implications across the lifespan. *Nat Commun.* (2020) 11:6285.

14. Stroup DF, Berlin JA, Morton SC, et al. Meta-analysis of observational studies in epidemiology: a proposal for reporting. Meta-analysis of observational studies in epidemiology (MOOSE) group. *JAMA.* (2000) 283:2008–12. doi: 10.1001/jama.283.15.2008

15. Moher D, Liberati A, Tetzlaff J, Altman DG, PRISMA Group. Preferred reporting items for systematic reviews and meta-analyses: the PRISMA statement. *Int J Surg.* (2010) 8:336–41. doi:

10.1016/j.ijsu.2010.02.007
16. Wardlaw JM, Smith EE, Biessels GJ, Cordonnier C, Fazekas F, Frayne R, et al. Neuroimaging standards for research into small vessel disease and its contribution to ageing and neurodegeneration. *Lancet Neurol.* (2013) 12:822–38. doi: 10.1016/S1474-4422(13)70124-8
17. Shi Y, Wardlaw JM. Update on cerebral small vessel disease: a dynamic wholebrain disease. *BMJ.* (2016) 1:83–92. doi: 10.1136/svn-016-000035
18. Szolnoki Z, Somogyvári F, Kondacs A, Szabó M, Fodor L, Bene J, et al. Specific APO E genotypes in combination with the ACE D/D or MTHFR 677TT mutation yield an independent genetic risk of leukoaraiosis. *Acta Neurol Scand.* (2004) 109:222–7. doi: 10.1046/j.1600-0404.2003.00218.x
19. Yoo JS, Ryu C-H, Kim YS, Kim H-J, Bushnell CD, Kim HY. Homocysteinemia is associated with the presence of microbleeds in cognitively impaired patients. *J Stroke Cerebrovas Dis.* (2020) 29:105302. doi: 10.1016/j.jstrokecerebrovasdis.2020.105302
20. Choi BO, Kim NK, Kim SH, Kang MS, Lee S, Ahn JY, et al. Homozygous C677T mutation in the MTHFR gene as an independent risk factor for multiple small-artery occlusions. *Thromb Res.* (2003) 111:39–44. doi: 10.1016/j.thromres.2003.08.022
21. Feng C, Yang Y, Yang S, Tu X, Wang Y, Song Y, et al. Effect of gene–gene and gene–environment interaction on the risk of first-ever stroke and poststroke death. *Mol Genet Genomic Med.* (2019) 7:846. doi: 10.1002/mgg3.846
22. Kawamoto R, Kohara K, Oka Y, et al. An association of 5,10-Methylenetetrahydrofolate Reductase (MTHFR) gene polymorphism and ischemic stroke. *J Stroke Cerebrovas Dis.* (2005) 14:67–74. doi: 10.1016/j.jstrokecerebrovasdis.2004.12.003
23. Kohara K, Fujisawa M, Ando F, Tabara Y, Niino N, Miki T, et al. MTHFR gene polymorphism as a risk factor for silent brain infarcts and white matter lesions in the Japanese general population. *Stroke.* (2003) 34:1130–5. doi: 10.1161/01.STR.0000069163.02611.B0
24. Li M, Fu B, Dong W. Correlations between plasma homocysteine and MTHFR gene polymorphism and white matter lesions. *Folia Neuropathol.* (2018) 56:301–7. doi: 10.5114/fn.2018.80863
25. Luo M, Li J, Sun X, Lai R, Wang Y, Xu X, et al. Interactions among candidate genes selected by meta-analyses resulting in higher risk of ischemic stroke in a Chinese population. *PLoS ONE.* (2015) 10:e0145399. doi: 10.1371/journal.pone.0145399
26. Mao X, Han L. The relationship of methylenetetrahydrofolate reductase gene C677T polymorphism and ischemic stroke in Chinese Han population. *Ann Clin Lab Sci.* (2018) 48:242–7.
27. Notsu Y, Nabika T, Park HY, Masuda J, Kobayashi S. Evaluation of genetic risk factors for silent brain infarction. *Stroke.* (1970) 30:1881–6. doi: 10.1161/01.STR.30.9.1881
28. Pavlovic AM, Pekmezovic T, Obrenovic R, Novakovic I, Tomic G, Mijajlovic M, et al. Increased total homocysteine level is associated with clinical status and severity of white matter changes in symptomatic patients with subcortical small vessel disease. *Clin Neurol Neurosurg.* (2011) 113:711–5. doi: 10.1016/j.clineuro.2011.07.004
29. Shen C, Zhang W, Sun K, Ling MX, Shen CC, Liu WG, et al. Interaction of genetic risk factors confers higher risk for thrombotic stroke in male Chinese: a multicenter case-control study. *Ann Hum Genet.* (2007) 71:620–9. doi: 10.1111/j.1469-1809.2007.00364.x

30. Szolnoki Z, Maasz A, Magyari L, Horvatovich K, Farago B, Somogyvari F, et al. The combination of homozygous MTHFR 677T and angiotensin II type-1 receptor 1166C variants confers the risk of small-vessel-associated ischemic stroke. *J Mol Neurosci.* (2007) 31:201–7. doi: 10.1385/JMN:31:03:201

31. Yeh P-S, Lin H-J, Li Y-H, Lin K-C, Cheng T-J, Chang C-Y, et al. Prognosis of young ischemic stroke in Taiwan: impact of prothrombotic genetic polymorphisms. *Thromb Haemost.* (2004) 92:583–9. doi: 10.1160/TH04-03-0099

32. You M, Zhou X, Yin W, Wan K, Zhang W, Li C, et al. The influence of MTHFR polymorphism on gray matter volume in patients with amnestic mild cognitive impairment. *Front Neurosci.* (2021) 15:778123. doi: 10.3389/fnins.2021. 778123

33. Chen L, Wu C, Dong Z, Cao S, Ren N, Yan X. Methylenetetrahydrofolate reductase polymorphisms and elevated plasma homocysteine levels in small vessel disease. *Brain Behav.* (2023) 13:2960. doi: 10.1002/brb3.2960

34. Szolnoki Z, Szaniszlo I, Szekeres M, Hitri K, Kondacs A, Mandi Y, et al. Evaluation of the MTHFR A1298C variant in leukoaraiosis. *J Mol Neurosci.* (2012) 46:492–6. doi: 10.1007/s12031-011-9621-4

35. Li T, Huang Y, Cai W, Chen X, Men X, Lu T, et al. Age-related cerebral small vessel disease and inflammaging. *Cell Death Dis.* (2020) 11:932. doi: 10.1038/s41419-020-03137-x

36. Martinez SS, Smith KJ. Understanding a role for hypoxia in lesion formation and location in the deep and periventricular white matter in small vessel disease and multiple sclerosis. *Clin Sci (Lond).* (2017) 131:2503–24. doi: 10.1042/CS20170981

37. Li S, Li G, Luo X, Huang Y, Wen L, Li J. Endothelial dysfunction and hyperhomocysteinemia-linked cerebral small vessel disease: underlying mechanisms and treatment timing. *Front Neurol.* (2021) 12:736309. doi: 10.3389/fneur.2021.736309

38. Rozen R. Genetic predisposition to hyperhomocysteinemia: deficiency of methylenetetrahydrofolate reductase (MTHFR). *Thromb Haemost.* (1997) 78:523–6. doi: 10.1055/s-0038-1657581

39. Ottavi TP, Pepper E, Bateman G, Fiorentino M, Brodtmann A. Consensus statement for the management of incidentally found brain white matter hyperintensities in general medical practice. *Med J Aust.* (2023) 219:278–84. doi: 10.5694/mja2.52079

40. Hong ED, Taylor WD, McQuoid DR, Potter GG, Payne ME, Ashley-Koch A, et al. Influence of the MTHFR C677T polymorphism on magnetic resonance imaging hyperintensity volume and cognition in geriatric depression. *Am J Geriatr Psychiatry.* (2009) 17:847–55. doi: 10.1097/JGP.0b013e3181aad5b2

41. Li Z, Wu X, Huang H, Xu F, Liang G, Lin C, et al. MTHFR C677T polymorphism and cerebrovascular lesions in elderly patients with CSVD: a correlation analysis. *Front Genet.* (2022) 13:987519. doi: 10.3389/fgene.2022.987519

42. Rutten-Jacobs LCA, Traylor M, Adib-Samii P, Thijs V, Sudlow C, Rothwell PM, et al. Association of MTHFR C677T genotype with ischemic stroke is confined to cerebral small vessel disease subtype. *Stroke.* (2016) 47:3. doi: 10.1161/STROKEAHA.115.011545

43. Rajagopalan P, Jahanshad N, Stein JL, Hua X, Madsen SK, Kohannim O, et al. Common folate gene variant, MTHFR C677T, is associated with brain structure in two independent cohorts of people with mild cognitive impairment. *Neuroimage Clin.* (2012) 1:179–87. doi:

10.1016/j.nicl.2012.09.012

44. Fan H, Yang S, Li Y, Yin J, Qin W, Yang L, et al. Assessment of homocysteine as a diagnostic and early prognostic biomarker for patients with acute lacunar infarction. *Eur Neurol.* (2018) 79:54–62. doi: 10.1159/000484893

45. Spence JD. Homocysteine lowering for stroke prevention: unravelling the complexity of the evidence. *Int J Stroke.* (2016) 11:744–7. doi: 10.1177/1747493016662038

46. Wang X-B, Dong H, Qiu Y-G, Lou C-C, Huang D-Y, Zhang J, et al. Nomogram based on clinical and brain computed tomography characteristics for predicting more than 5 cerebral microbleeds in the hypertensive population. *Front Neurol.* (2022) 13:955378. doi: 10.3389/fneur.2022.955378

47. Zhang DD, Cao Y, Mu JY, Liu YM, Gao F, Han F, et al. Inflammatory biomarkers and cerebral small vessel disease: a community-based cohort study. *Stroke Vasc Neurol.* (2022) 7:302–9. doi: 10.1136/svn-2021-001102
